# Variation and Variability in Skeletal Ossification of the Gray Short-tailed Opossum, *Monodelphis domestica*

**DOI:** 10.1093/iob/obae024

**Published:** 2024-07-03

**Authors:** Z S Morris, M W Colbert, T B Rowe

**Affiliations:** Ja ckson School of Geosciences, The University of Texas at Austin, Austin, TX 78712, USA; Dinosaur Institute, Natural History Museum of Los Angeles County, Los Angeles, CA 90007, USA; Ja ckson School of Geosciences, The University of Texas at Austin, Austin, TX 78712, USA; University of Texas High‐Resolution X‐Ray CT Facility, The University of Texas, Austin, TX 78712, USA; Ja ckson School of Geosciences, The University of Texas at Austin, Austin, TX 78712, USA; University of Texas High‐Resolution X‐Ray CT Facility, The University of Texas, Austin, TX 78712, USA

## Abstract

By reconstructing and comparing the sequence of ontogenetic (embryonic development and post‐natal growth) events across species, developmental biologists have gained unique insights into the key processes underlying the evolution of modern lineages and their extinct relatives. However, despite the importance of intraspecific variation to evolutionary transformation and lineage divergence, variation in the sequence of developmental events is seldom acknowledged. Thus, how much variation or variability should be expected during ontogeny remains poorly understood and it is an open question to what extent it impacts interspecific comparisons of developmental patterns. To address this crucial question, we studied the skeletal development of the important biomedical and developmental model organism, *Monodelphis domestica*. We investigated cranial, forelimb, and hindlimb elements using ontogenetic sequence analysis (OSA) to quantify and assess the full range of variation and variability in the sequence of ossification. Our study documented that previously unrecognized variation exists during *M. domestica* ontogeny—with over 5000 sequences for the full 92 event analysis. Further, OSA revealed unexpectedly high variability (i.e., the propensity to express variation) in the sequence of ossification for the skull and across the entire skeleton. Reconstructed modal sequences were generally in agreement with previously recognized patterns, including earlier ossification of the facial skeleton and a slight offset between forelimb and hindlimb development. However, the full range of variation shows that the majority of specimens in our analysis followed developmental trajectories distinct from those recovered by prior studies. This level of variation is quite remarkable and demonstrates the importance of assessing intraspecific ontogenetic variation. By quantifying sequence polymorphism and studying how developmental variation and variability differ among species, we can clarify more precisely how developmental patterns differ among species and gain insights into how ontogeny itself evolves.

## Introduction

Embryological researchers have discussed the appropriate methods for reconstructing ontogenetic sequences for over 150 years (Haeckel 1866; de Beer 1930; Hennig 1966; Gould 1977). Traditional methods involve elucidating typified stages of ontogeny by observation of several individual semaphoronts—a particular period of maturity during ontogeny (Hennig 1966). Such investigations have resulted in a tremendous wealth of information about development, from the early differentiation and morphogenesis (Marcucio et al. 2015; Dash and Trainor 2020; McQueen and Towers 2020) to later patterns of ossification for all major vertebrate lineages from ray-finned fish (e.g., de Jong et al. 2009) and amphibians (e.g., Hanken and Hall 1984; Harrington et al. 2013) to reptiles (e.g., Rieppel 1993; Maisano 2002; Sánchez‐Villagra et al. 2009), birds (e.g., Nakane and Tsudzuki 1999; Maxwell 2008; Maxwell and Larsson 2009), and mammals (e.g., Clark and Smith 1993; Sánchez‐Villagra et al. 2002; Wilson et al. 2010). The development of methods for interspecific comparisons of these patterns (e.g., Nunn and Smith 1998; Smith 2001; Jeffery et al. 2002, 2005; Harrison and Larsson 2008) has enabled new investigations of developmental constraints and reconstructions of the evolution of ontogenetic sequences (e.g., Smith 1997; White et al. 2023). Despite the difficulties associated with collecting ontogenetic series for non-model organisms, the last two decades have seen several groups build new developmental datasets for a tremendous diversity of mammalian clades (e.g., Sánchez‐Villagra et al. 2008; Weisbecker et al. 2008; Sears 2009; Hautier et al. 2011; Koyabu et al. 2011; Weisbecker 2011). These studies have provided critical insights into the placental-marsupial divergence and directly address the relative importance of intrinsic developmental processes and extrinsic selective pressures in creating the disparity observed within Theria. However, the extent to which intraspecific developmental variation may affect reconstructions of the evolution of ontogenetic patterns remains unclear.

The variation within species during development has long been recognized and was a major reason for the development of typified staging tables by 19th century developmental biologists (Hopwood 2007). Although nearly all developmental studies recognize some degree of intraspecific variation and several studies of ossification have explicitly measured it (e.g., Mabee et al. 2000; Moore and Townsend 2003; Wilson et al. 2010; Mitgutsch et al. 2011), the most commonly applied methods for creating and comparing sequences among species do not readily enable inclusion of such variation. This is remarkable because analyses routinely recover significant variation across all regions of the skeleton (Mitgutsch et al. 2011), with up to 50% of events being variable (Wilson et al. 2010). Just as phenotypes can be polymorphic within a population, the existence of sequence polymorphism (i.e., intraspecific ontogenetic sequence variation; Garn et al. 1966) means that there is a population of ontogenetic sequences from which traditional methods are sampling. Understanding this variation is crucial as it enables identification of which events are well-resolved enough for comparison (e.g., Mabee et al. 2000; Moore and Townsend 2003). Further, species may differ not only in the sequence of events in ontogeny, but also differ in the range of sequence variation and variability—the frequency with which variant patterns are expressed (*sensu*Wagner and Altenberg 1996). To properly assess how ontogenetic sequences evolve, it is fundamental that we understand not only a typical ontogenetic pattern but also the variation and variability in that population.

Ontogenetic sequence analysis (OSA) is a method of reconstructing ontogeny that explicitly quantifies sequence polymorphism to understand the full population of developmental patterns within species (Colbert 1999; Colbert and Rowe 2008). This method is similar to parsimony based phylogenetic analyses, but instead of scoring different taxa for characters, semaphoronts are scored for developmental events. This reveals a network of semaphoronts with all identifiable ontogenetic sequences such that all individuals are part of the same developmental population. The treatment of all specimens as potentially unique semaphoronts distinguishes OSA from other methods of ontogenetic sequence reconstruction and is critically important for recovering sequence polymorphism. Previous analyses using OSA (e.g., Colbert and Rowe 2008; de Jong et al. 2009; Olori 2013; Griffin 2018) have found substantial, if variable, levels of sequence polymorphism during skeletal development in a variety of vertebrates. However, it remains unknown what impact sequence variation and variability may have on comparative studies of mammalian developmental patterns. Our study addresses this fundamental question by assessing levels of variation in the sequence of skeletal ossification of the gray short-tailed opossum, *Monodelphis domestica* (Wagner 1842), an important model system in developmental and biomedical research (VandeBerg and Robinson 1997; Keyte and Smith 2008; Dowling et al. 2016; Kiyonari et al. 2021). The sequence of skeletal ossification for *M. domestica* has been studied using traditional methods (Clark and Smith 1993) and been included in every study which compares the ossification patterns of marsupials and placentals (e.g., Sánchez‐Villagra et al. 2008; Weisbecker et al. 2008; Sears 2009; Hautier et al. 2011; Koyabu et al. 2011; Weisbecker 2011), which makes *M. domestica* the ideal subject for our analysis. By quantifying how variation is structured across ontogeny and among anatomical regions, our analysis assesses which events may be more reliable markers of maturity and identifies developmental patterns that are consistent across the entire population. Our study further explores the potential impact this variation and variability has on comparative studies by contrasting the results of OSA to previously published analysis of *M. domestica*. Our analysis reveals the importance of sequence polymorphism and how direct comparisons of ontogenetic variation have the potential to provide crucial insights into the factors underlying developmental evolution.

## Materials and methods

A total of 38 cleared‐and‐stained specimens of *M. domestica* of known age (in postnatal days) from The University of Texas at Austin, Vertebrate Paleontology Laboratory were used in this study (Table 1). The *M. domestica* growth series was acquired from the Southwest Foundation for Biomedical Research (now called Texas Biomedical Research Institute), in San Antonio, Texas which maintained an NIH-funded breeding colony of *M. domestica* for biomedical research. All animals were reared under the same conditions and genetic outcrossing was done to alleviate concerns about inbreeding and genetic variability (Fadem et al. 1982). T.B. Rowe, in consultation with the staff, commissioned a time-calibrated growth series that was assembled between 1988 and 1992 (NSF‐BSR-89–58092, NSF-EAR-1561622). Three to five individuals (both male and female) each from days 0–30, 33, 36, 39, 42, 45, 48, 54, 57, 65, 70, 75, 80, 85, 90, and “adults” (“retired breeders”). Additional specimens were collected opportunistically, as some individuals died in captivity and were made available for the project. Approximately, 100 specimens dating from day 0 through “adults” were cleared and double stained by Dr. Rafael de Sá, using Alizarin red‐S to stain bone and Alcian blue to stain cartilage (following Hanken and Wassersug 1981). Additional individuals from postnatal days 0, 1, 10, 15, 27, and 36 were serially sectioned using conventional histological techniques and stained with azocarmine and dried skeletons dating from postnatal day 27 through adults were prepared, but not included in this analysis.

**Table 1:** List of the 38 specimens of *Monodelphis domestica* used in this analysis, with collection ID number, specimen code, ages in post-natal days, and maturity score given for each specimen

Collection number	Specimen code	Age (in post-natal days)	Maturity score
TMM-7612	0b	0	0
TMM-7615	1a	1	0
TMM-7616	1b	1	15
TMM-7619	1d	1	14
TMM-7623	2c	2	15
TMM-7624	2d	2	24
TMM-7625	3a	3	14–16
TMM-7626	3b	3	28–35
TMM-7627	3c	3	33–35
TMM-7628	day 3	3	25
TMM-7629	4a	4	24
TMM-7633	5b	5	34
TMM-7635	day 6	6	19–47
TMM-7636	7a	7	41
TMM-7637	7b	7	42
TMM-7638	7c	7	48
TMM-7639	day 7	7	35
TMM-7640	8a	8	47–48
TMM-7641	8b	8	47
TMM-7642	8c	8	43
TMM-7643	day 8	8	49
TMM-7644	9a1	9	53–55
TMM-7645	9a2	9	50
TMM-7646	9b	9	53
TMM-7647	10a	10	55
TMM-7649	11a	11	52–53
TMM-7650	11b	11	54
TMM-7654	13a	13	65
TMM-7659	15a	15	74
TMM-7660	15b	15	72
TMM-7662	15d	15	73
TMM-7663	15e	15	61–63
TMM-7664	15f	15	67
TMM-7665	15g1	15	72
TMM-7667	16a	16	72
TMM-7671	17b	17	73
TMM-7679	20a	20	76
TMM-7684	21b	21	76–77

*Note:* Ambiguity in event scores resulted in a range of potential maturity scores in some cases.

Rather than using age or size, which do not perfectly correlate with developmental progress, as proxies for maturity, we used OSA to reconstruct all potential ossification sequences which could have resulted in this observed dataset (Colbert and Rowe 2008). Although other methods exist, including parsimony based ones (e.g., Brochu 1996; Carr 2020), only OSA further enables the quantification of the full range of variation and variability, inclusion of missing or uncertain data, and assessment of how well the progression of skeletal ossification correlates with age. Each specimen was individually scored for 92 ontogenetic events (Table S1), quantifying the ossification of 25 cranial and 67 appendicular elements (see supplementary methods). The initial ossification of each element was scored based on visual observation of Alizarin red staining or significant texturing (e.g., spicules of bone) under light microscopy. We initially scored all paired elements separately, but in our dataset, there were no individuals for which only one elements was observed to have ossified and, therefore, all paired events were scored jointly in the final dataset. The lack of asymmetry in ossification is likely an artifact of our sampling. Throughout this manuscript, we follow the skeletal nomenclature for generalized tetrapods as this has been applied in prior studies (e.g., Clark and Smith 1993) and use the plural for all elements which ossify initially as paired elements. One exception is the interparietal as, similar to Clark and Smith (1993), we did not find evidence of separate “tabular” or “post-parietal” ossification centers. Likely reflecting our inability to sample the appropriate semaphoronts, the interparietal was instead detected as an osseous matrix which spanned the initial “post-parietal” ossifications (*sensu*Koyabu et al. 2012). In preparation for OSA, the matrix of event‐scores for all specimens was transformed into semaphoront‐event matrices by combining specimens with equivalent event scores. To understand whether patterns of variation differed across the skeleton, we performed OSA on cranial, forelimb, and hindlimb partitions, in addition to the combined dataset. Additionally, these partitioned analyses enabled more precise resolution of individual ossification events in our sequences, as this is determined by the ratio of semaphoronts to events in any developmental study (Colbert 1999).

To reconstruct ontogenetic sequences for OSA we used the parsimony algorithm implemented in Paup* 4.10b (Swofford 2002). Sets of “sequence maps” (i.e., phylogenetic trees) were recovered for all analyses by conducting two analyses, first with the least mature semaphoront (all events scored 0) as the “outgroup” and then with a hypothetical fully mature semaphoront (all events scored 1) as the “outgroup.” By focusing on the events of interest, rather than the age of individuals, OSA reveals all necessary sequences and the order in which all events occur across the population of sequences. All characters were set as irreversible under a heuristic search algorithm which added semaphoronts randomly, using tree bisection and reattachment search parameters for 20,000 replicates each. The irreversibility of events is fundamental to OSA as once an ontogenetic event occurs it cannot be undone. Although it is possible for a center of ossification to form and then be destroyed, this would be considered a secondary ontogenetic event and not a reversal, as the initial ossification did occur. If secondary loss cannot be distinguished from primary absence, then that event is not amenable to OSA. The raw OSA semaphoront‐event matrices (as nexus files) can be found in the online supplements. The sets of “sequence maps” generated were then imported into MacClade 4.08a (Maddison and Maddison 2005) and characters were traced as irreversible. The character tracing data were used to construct a full network of ontogenetic sequences for the population (following Colbert and Rowe 2008) with semaphoronts arranged by maturity score (i.e., the total number of ossification events an individual presents) and connected by sequence segments reconstructed by OSA. Thus, the OSA network contains all developmental sequences which must be inferred such that all specimens connect to both the least and most mature conditions. This includes not only the semaphoronts observed in the dataset, but also unobserved, yet necessary, intermediate semaphoronts. These inferred semaphoronts represent testable hypotheses generated using OSA, which additional sampling can target as a test of the validity of the network. There is a finite set of theoretically possible sequences expressed as the factorial of binary events considered by OSA. For the 92 events in the total dataset, there is a hypothetical 92 dimensional hypervolume comprised of 1.244 × 10^142^ fully resolved ossification sequences, of which some portion was occupied by *M. domestica*. With a large enough sample size, likelihood statistics can be applied in this theoretical multidimensional sequence space. However, with 38 specimens, our analysis did not sample heavily enough for the application of likelihood values and the determinations of key features were based on raw number of specimens instead of true likelihood statistics.

Once the network has been constructed, key features like the modal sequence, total sequence polymorphism, and sequence variability can be characterized. The modal sequence—the ontogenetic sequence which appears to be the most commonly followed by the sampled specimens—was calculated using a frequency‐weighted value based on the number of specimens representing each semaphoront. When two scored individuals had the same event‐scores, then their values were combined (i.e., a weight of 2). Similarly, when a single individual possessed uncertain scores for events, their value was split evenly between potential semaphoronts. For the purposes of frequency calculations, semaphoronts that were reconstructed by OSA but not observed in the dataset receive no weight. For each analysis, the total number of reconstructed sequences captured the range of variation, or sequence polymorphism, for that dataset. The estimated level of sequence polymorphism is a consequence of incompatible event scores among semaphoronts that reveal multiple developmental patterns must exist. Sequence variability, in contrast, was characterized by comparing the frequency‐weight of the modal sequence(s) to the average weight across all sequences. Thus, sequence polymorphism is the amount of ontogenetic variation present, while sequence variability is a measure of how frequent these variant sequences are in the sampled population. Thus, variation is reflected in how many sequences are recovered, while variability is reflected in how evenly distributed individuals are across the modal and non-modal sequences. In addition to the network diagram, the relative timing of individual ossification events across all sequences were investigated—revealing important insight into the variability or uncertainty in the timing of certain events and whole partitions in the combined dataset. This is referred to as the “sequence position” of an event, and corresponds to the range of maturity scores over which the event is reconstructed to occur. Critically, events which co‐occur on sequence segments should not be considered coincident, but simply occurring in an unresolved sequence due to a lack of intermediate semaphoronts.

## Results

### Whole skeleton OSA

**Fig. 1:**
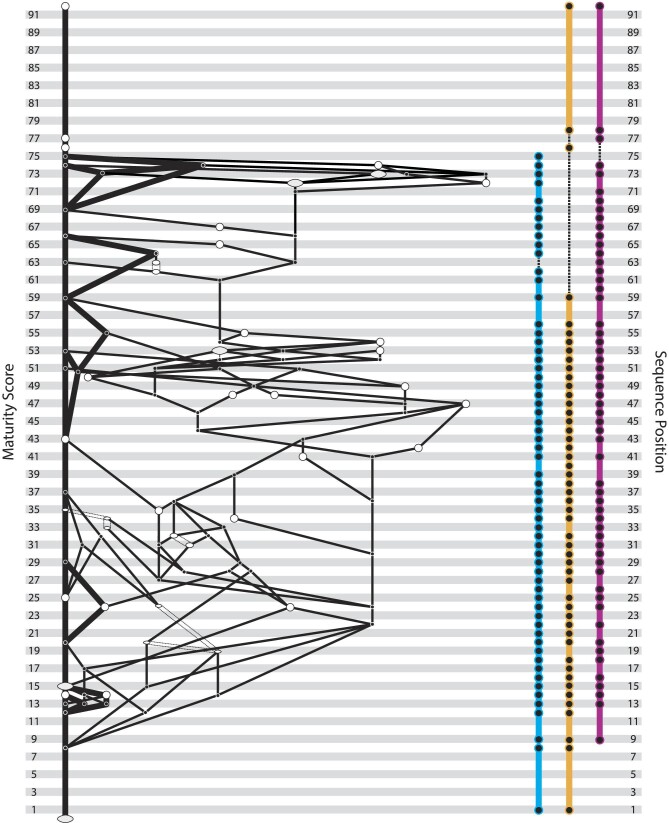
Ontogenetic sequence analysis (OSA) network diagram of the 5128 unique parsimonious ossification sequences for the whole skeleton (left) alongside the sequence position of cranial (blue), forelimb (yellow), and hindlimb (pink) element ossifications (right). Observed semaphoronts are represented by open ellipses, with width proportional to frequency support, and unobserved semaphoronts reconstructed by OSA are represented by filled nodes. The modal sequences are shown by bold segments. Ambiguity resulted in semaphoronts with multiple possible maturities represented by double-dashed lines. Specimens and events shown in Fig. S1.

**Fig. 2:**
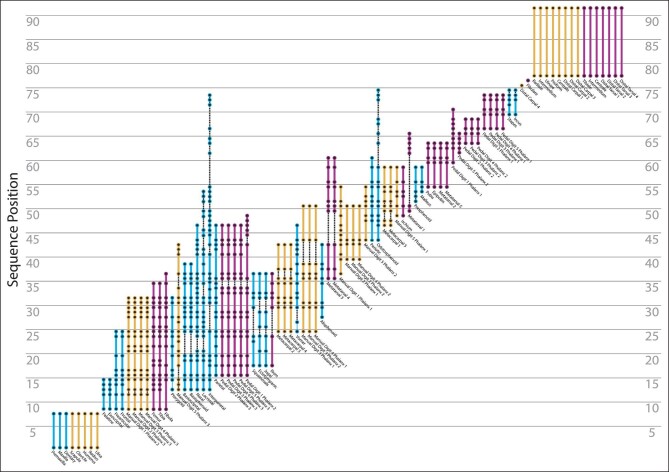
Diagram of the sequence position for all ossification events in the whole skeleton OSA, with cranial (blue), forelimb (yellow), and hindlimb (pink) partitions highlighted. Solid lines represent sequence segments in which individual events were resolved. Dashed lines represent events resolved at different positions in alternate sequences but not at the intervening positions.

**Fig. 3:**
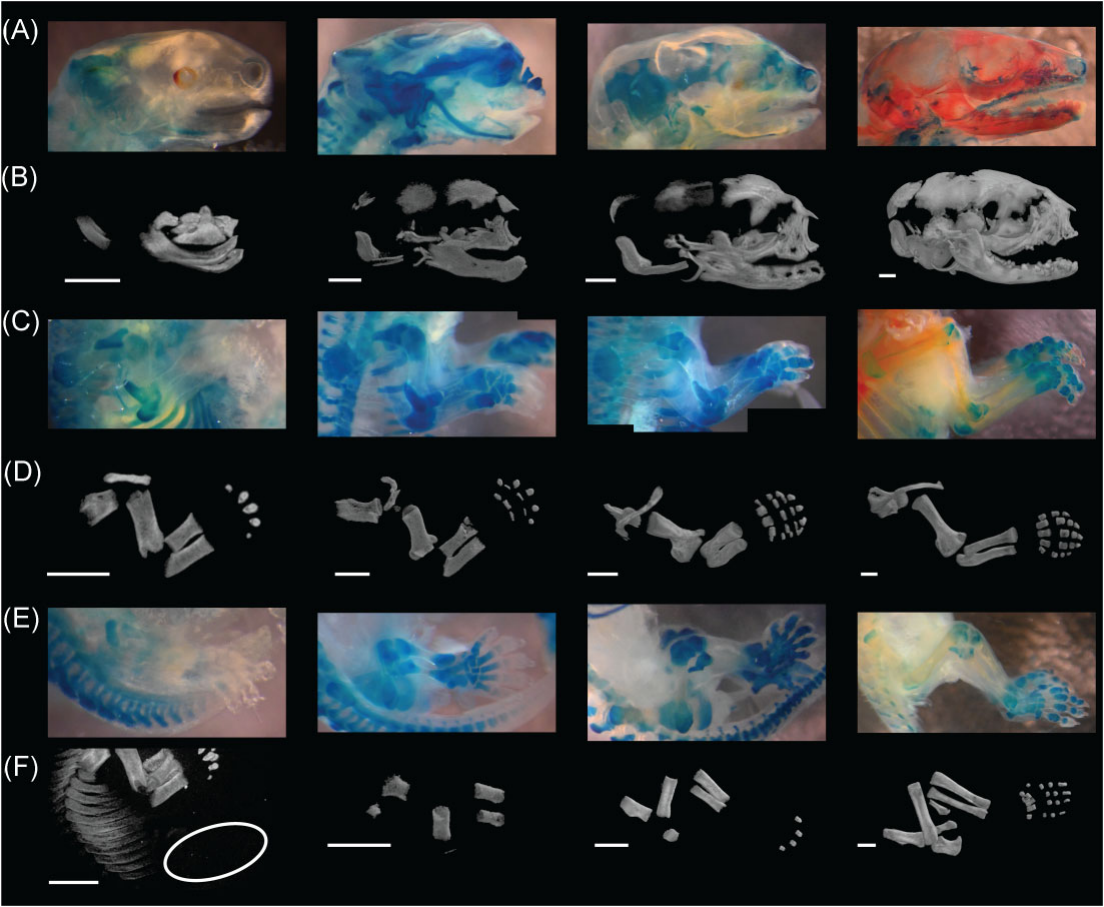
Overview of the general trends in the ossification of the skull (A, B), forelimb (C, D), and hindlimb (E, F) in *Monodelphis domestica*. Microscope images of the cleared and stained specimens (A) TMM-7621 (day 2), 7630 (day 4), 7665 (day 15), and 7682 (day 20); (C) TMM-7615 (day 1), 7635 (day 6), 7642 (day 8), and 7684 (day 21); (E) TMM-7615 (day 1), 7642 (day 8), 7665 (day 15), and 7684 (day 21)) are shown in comparison to rendered volumes from CT scans (B, D, F; TMM-7616 [day 1], 7633 [day 5], 7665 [day 11], and 7679 [day 20]). The unossified hindlimb of TMM-7616 is shown in the circled region of F. Scale bars represent 1 mm.

Ontogenetic sequence analysis of the initiation of ossification across the whole skeleton of *M. domestica* (92 events) recovered 5,128 partially resolved ontogenetic sequences (Fig. 1, Fig. S1). The 38 scored specimens represented between 33 and 39 unique semaphoronts because four specimens, TMM‐7626 (3b), 7627 (3c), 7635 (day 6), and 7663 (15e), could not be clearly scored for some events and, therefore, possessed a range of potential maturity scores. We did not recover a single modal sequence, but rather a set of 144 sequences with equal frequency weight (9.333 specimens). However, OSA recovered substantial sequence polymorphism and sequence variability, as the modal sequences included only a single additional specimen than the next highly weighted set of sequences. For the skull, the facial skeleton was the first to initiate ossification, followed closely by portions of the palate and skull base before the cranial vault elements, and finally more internal braincase and middle ear elements (Fig. 2). For both forelimb and hindlimb partitions, the girdle, stylopodial, and zeugopodial elements ossified first, followed by the most distal phalanges, then the metapodials and more proximal phalanges, and finally the carpals or tarsals. The most frequent sequences in general recovered the ossification of the anterior portion of the skull (facial region) and the major forelimb elements as the first events in skeletal ontogeny (Fig. 3). Hindlimb features were slightly delayed in the most frequent ontogenetic sequences, but were not meaningfully delayed from the onset of forelimb ossification (Figs. 1 and 3). However, the precise timing of closely occurring events were polymorphic and no single sequence could result in all of the observed specimens in our analysis.

Across the entire skeleton, we found considerable sequence polymorphism in the timing of ossification. Outside of the first and final three sequence segments, all other events occurred at distinct points across multiple ontogenetic sequences (Fig. 2). The events with the greatest range of sequence positions (i.e., occurring at different maturity scores) were predominantly found in the skull, with the interparietal ossifications (cf. “post-parietals” *sensu*Koyabu et al. 2012), orbitosphenoids, and lacrimals being the most variable. However, other portions of the basicranium and the distal most phalanges in both the forelimb and hindlimb also showed extensive variation across sequences. Only the fibularia (paired fibulare) and fourth distal carpals were simultaneously reconstructed to a single sequence segment and precisely resolved as individual events. However, this is due in part to sampling bias, as some individual sequences did resolve other events to a single point in ontogeny and certain sets of events were simply unresolvable given our data. No single sequence was represented by more than ten specimens and most sequences included only four observed specimens. In fact, the majority of sampled specimens (28 out of 38) could not have followed the modal ossification sequences. Thus, *M. domestica* not only possesses high variation in the sequence of ossification (i.e., sequence polymorphism), but also possesses high sequence variability (i.e., variation is highly expressed). The lack of a single obvious modal sequence in our analysis is further evidence of this variation and variability in the sequence of ossification across the whole skeleton.

### Cranial OSA

**Fig. 4:**
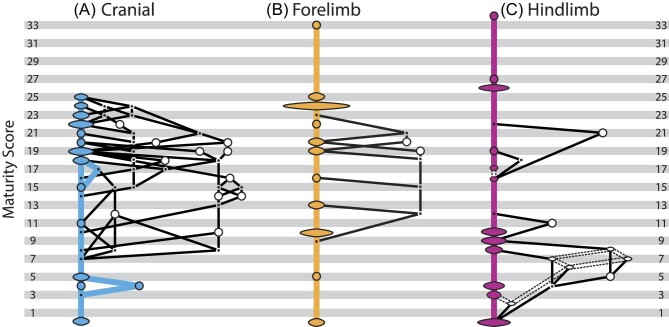
Ontogenetic sequence analysis (OSA) network diagrams for cranial (A), forelimb (B), and hindlimb (C) partitions. Specimens and events are shown in Figs. S2–S4.

The onset of ossification for cranial elements showed more intraspecific variation than any other data partition, including the whole skeleton OSA. The cranial matrix generated over 22,000 “sequence maps” just for the initial analysis using the least mature individual as the “outgroup.” Given the vast number of “sequence maps” recovered, it was not feasible to visually inspect each for evidence of sequence polymorphism and the range of variation recovered here most likely under‐represents the real amount recovered. As many “sequence maps” as possible were randomly selected and inspected, until additional sequences were no longer recovered. When completed, we recovered over 924 parsimonious ontogenetic sequences (Fig. 4A, Fig. S2) for the 25 cranial ossification events based on a total of 25 observed semaphoronts. The reduced number of semaphoronts compared with the whole skeleton OSA reveals that several individuals possessed identical cranial event scores, but distinct forelimb and hindlimb scores. This suggests that the whole skeleton OSA may be better able to break up these events and provide relatively more precise sequence placements.

**Fig. 5:**
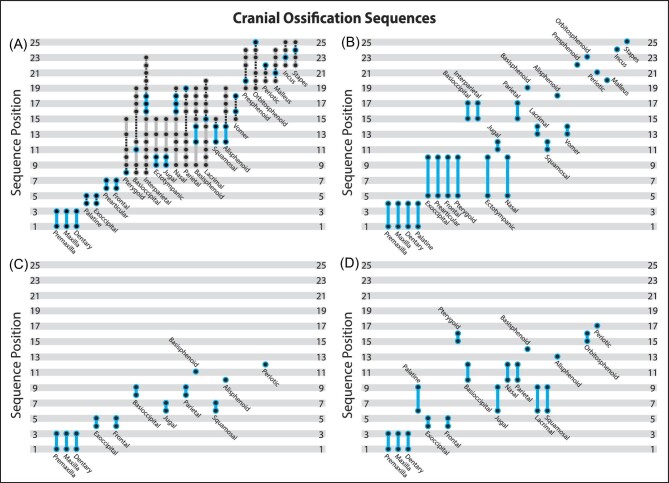
Comparison of the sequences of cranial ossification based on OSA (A), Clark and Smith 1993 (B), Nunn and Smith 1998 (C), and Hautier et al. 2011 (D). Sequences are ordered by first appearance in our study, with the modal sequences highlighted in blue and alternative sequences in gray. Dashed lines represent events resolved at different positions in alternate sequences but not at those specific positions. Nunn and Smith 1998 (C) and Hautier et al. 2011 (D) did not study all 25 ossification events.

**Fig. 6:**
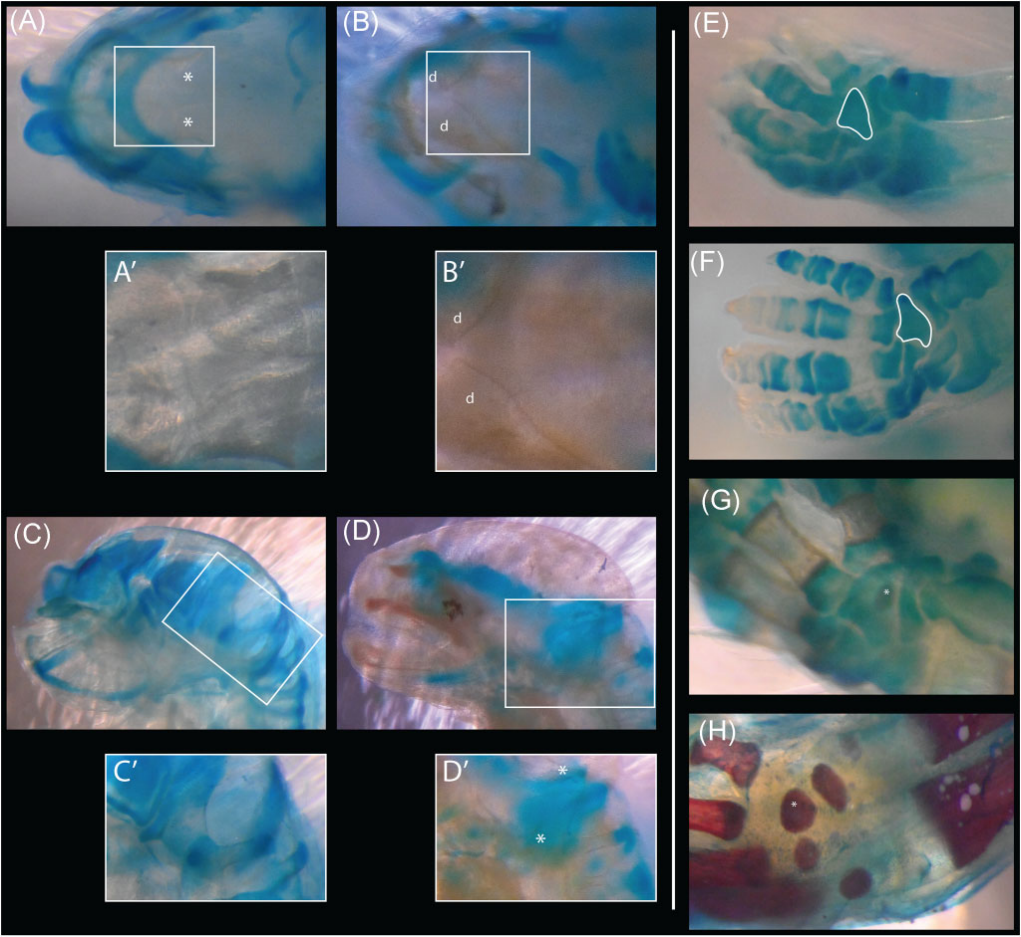
Comparisons highlighting both intraspecific variation and invariability in the timing of ossification. TMM-M-7619 (day 1) and 7625 (day 3) demonstrate key differences in the timing of ossification of the palatines (A, B) and exoccipitals (C, D), despite possessing identical maturity scores. TMM-7619 shows clearly ossified palatines (A’) and lacks any staining or texturing of the exoccipitals (C’), while the inverse is apparent in TMM-7625 (B’, D’). In contast, the fourth distal carpal is always the first carpal element to ossify in *M. domestica*. The unossified state shown in the outlined elements in TMM-7644 (day 9) and 7664 (day 15), initial ossification in TMM-7684 (day 21), and the ossification of additional elements in TMM-7718 (day 29). Asterisks highlight less clear ossification centers and “d” labels the ossified dentaries in TMM-7625.

The cranial OSA resulted in no single most frequent sequence, but four distinct sequences with the highest frequency weight (26 specimens). These sequences recovered a pattern in which the premaxillae, maxillae, and dentaries ossified first, unresolved relative to one another. In half of the sequences, the palatines ossified next, followed by the exoccipitals, but in the other half the sequence of events was reversed (Fig. 5A). This difference was recovered because specimens TMM‐7619 (1d) and 7625 (3a) possessed equal maturity scores, but differed in the ossification states for these elements (Fig. 6A–D). Next, all sequences recovered the ossification of the prearticulars (synonymous with os goniale or gonial; Anthwal et al. 2013) and frontals in an unresolved sequence. In the modal sequences, the pterygoids were the next element to begin to ossify, followed by the ectotympanics and jugals in an unresolved sequence. The basioccipital was resolved next, at sequence position 11. Then, the squamosals, alisphenoids, and basisphenoid ossified, unresolved relative to each other. After these three events, the ossification of the lacrimals was resolved precisely at sequence position 15 in all four modal sequences. Subsequently, the modal sequences followed two different pathways: one in which the vomer ossified, followed by both the nasals and interparietal, and another in which the vomer began to ossify later. The last seven ossification events were individually resolved in the modal sequences in the order: parietals, presphenoids, mallei, periotics, incudes, stapedes, and finally orbitosphenoids. However, OSA recovered over 900 distinct non‐modal sequences due to 11 specimens which possessed event states incompatible with this pattern of ossification. Sequences diverged dramatically after the ossification of the prearticulars (gonials) and frontals, with TMM‐7628 (day 3), 7629 (4a), and 7635 (day 6) being the major sources of variation. Even well resolved events, like the ossification of the pterygoids are shown to be capable of occurring up to seven events later along other sequences. Thus, the cranial dataset shows similar levels of sequence variation and variability as the whole skeleton OSA.

Given our inability to assess all trees individually, it must be emphasized that this analysis may not reflect all sequences. However, OSA enables us to identify consistent features of cranial ontogeny. The premaxillae, maxillae, and dentaries were always the first three elements to ossify, followed by the palatines and exoccipitals in all sequences. Although the relative order of palatine and exoccipital ossification is variable, all sequences converged at a single semaphoront, represented by TMM‐7616 (1b) and 7623 (2c), which may reflect a developmental constraint on the subsequent ossification of the prearticulars (gonials) and frontals. After this point in ontogeny there was considerable variation among ossification sequences, particularly in the case of the ossification of the nasals, lacrimals, parietals, interparietal, basioccipital, and basisphenoid which occurred from sequence position eight through 22. These elements were similarly variable in the whole skeleton OSA. In the vast majority of the sequences recovered by OSA, the last three events to occur were the ossification of the incudes, stapedes, and orbitosphenoids, individually resolved. Therefore, across all of the variation observed these results are generally in agreement with the whole skeleton OSA: The facial skeleton was the first to initiate ossification, followed closely by portions of the palate and skull base before the cranial vault elements, and finally more internal braincase and middle ear elements (Fig. 3A and B).

### Forelimb OSA

**Fig. 7:**
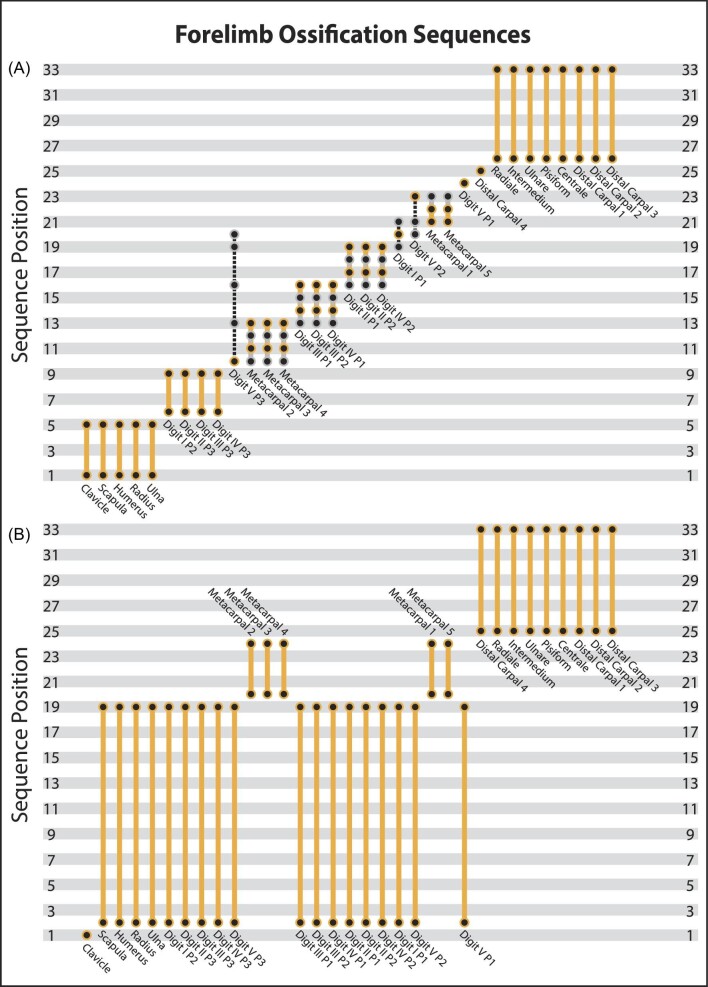
Comparison of the sequences of forelimb ossification based on OSA (A) and Weisbecker et al. 2008 (B). Sequences are ordered by first appearance in our study, with the modal sequences highlighted in yellow and alternative sequences in gray. Dashed lines represent events resolved at different positions in alternate sequences but not at those specific positions.

The forelimb partition included 12 observed semaphoronts for 33 events and OSA recovered a total of 14 ontogenetic sequences (Fig. 4B, Fig. S3). The single modal sequence was represented by 36 of the 38 specimens, reflecting a substantial reduction in reconstructed variation in forelimb ossification relative to cranial and whole skeleton OSAs. In the forelimb modal sequence, the scapulae, clavicles, humeri, radii, and ulnae were the first elements to ossify, but the relative sequence of these events was unresolved (Fig. 7A). Subsequently, the distal phalanges of digits I through IV ossified, prior to the distal phalanges of the fifth digits, which was resolved to a single sequence position (the 10th). Crucially, the precise placement of this event was only possible because OSA reconstructed an unobserved semaphoront just prior to this position. Intriguingly, this event is the most variable across the entire dataset as it occurred as the 10th, 13th, 16th, 19th, or 20th event despite being individually resolved in all sequences. Then, three successive segments of three events each occurred. First, metacarpals II, III, and IV ossified, followed by the first and second phalanges of the third digits and the first phalanges of the fourth digits, then the first and second phalanges of the second digits and the second phalanges of the fourth digits. The ossification of the most proximal phalanges of the first digits were next resolved to a single sequence position (the 20th), followed by metacarpals I and V in an unresolved sequence. The second phalanges of the fifth digits ossified next and were followed by the first phalanges, both of which were resolved to single sequence positions. Our dataset did not include specimens which helped to resolve the order of carpal ossification, except that the fourth distal carpals initiated ossification before all other wrist elements (Fig. 6E–H).

In all sequences the scapulae, clavicles, humeri, radii, and ulnae were first to ossify (Fig. 3C, D). The ossification of the first phalanges of the fifth digits, the fourth distal carpals, and all other carpals were the last three segments in all sequences reconstructed by OSA. Between these points in ontogeny, bounded by maturity scores 9 and 23, was where sequence polymorphism was concentrated. The major cause of variation among sequences was TMM‐7636 (7a), which lacked ossified distal phalanges on the fifth digits, despite having a maturity score of 19 and a well ossified rest of the forelimb. This single specimen is the source of the most sequence polymorphism and forced the ossification of the distal phalanges of the fifth digits to be recovered later and across more positions in ontogeny. TMM‐7643 (day 8) was also on a non‐modal sequence due to a lack of proximal phalanges of the first digits, while also possessing ossified second phalanges of the fifth digits. Thus, simply two specimens were needed to identify 13 additional ontogenetic sequences in the forelimb.

### Hindlimb OSA

The OSA of the hindlimb partition reconstructed 36 ossification sequences (Fig. 4C, Fig. S4) for the 34 scored events. There was greater ambiguity in the hindlimb events because two specimens, TMM‐7635 (day 6) and 7663 (15e), could not be confidently scored for some events which increased the number of potential semaphoronts from 13 to 19. This resulted in OSA identifying greater sequence polymorphism than for the forelimb, but still remarkably limited expressed sequence variability. The hindlimb modal sequence was weighted by all but three specimens, including both putative semaphoronts represented by TMM‐7663 and 1/6 of those for TMM‐7635. Thus, the additional non‐modal sequences were only represented by fewer than four specimens, suggesting that the expressed variability in ossification sequence is similarly lower in both limb partitions relative to cranial ossification.

**Fig. 8:**
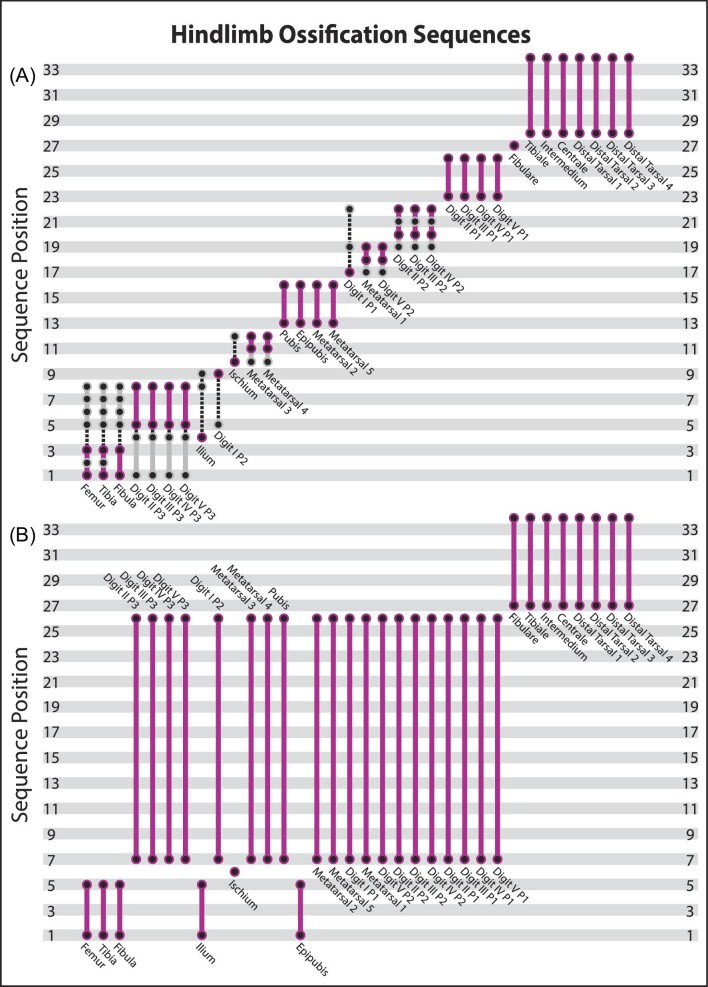
Comparison of the sequences of hindlimb ossification based on OSA (A) and Weisbecker et al. 2008 (B). Sequences are ordered by first appearance in our study, with the modal sequences highlighted in pink and alternative sequences in gray. Dashed lines represent events resolved at different positions in alternate sequences but not at those specific positions.

In the modal sequence, the first segment included the ossification of the femora, tibiae, and fibulae, unresolved relative to one another (Fig. 8A). Subsequent to this, the ilia ossified at sequence position 4. Then, the distal phalanges of digits II though V ossified. The ossification of the distal phalanges of the first digits occurred subsequently and was followed by the ossification of the ischia, both resolved to sequence positions 9 and 10, respectively. Then, both metatarsals III and IV ossified, followed by the ossification of the pubes, epipubes, and metatarsals II and V. After this point, the first phalanges of the first digits ossified at sequence position 17. Then, the first metatarsals and the second phalanges of the fifth digits ossified prior to the second phalanges of digits II, III, and IV. The timing of the onset of ossification in the first phalanges of the first digits relative to the above pattern is what creates variation in this portion of sequence space (between sequence positions 13 and 22). After this region, all sequences in the entire analysis were identical. The first phalanges of digits II, III, IV, and V ossified next, unresolved relative to one another. The ossification of the fibularia, which was the only event to be fully resolved in the hindlimb OSA, occurred just prior all other tarsals, at sequence position 27. Our dataset was unable to resolve the timing of ossification for the other tarsals in a similar pattern as for the forelimb.

In contrast to the other partitions, the hindlimb shows greater sequence polymorphism in the early ossification events. Although due in part to the ambiguity introduced by TMM7635 (day 6), this pattern appears to reflect a real pattern as TMM‐7628 (day 3) could be scored for all events and still falls along non‐modal sequences early in ontogeny. This early variation reflects whether distal phalanges ossify after the femora, tibiae, fibulae, and ilia (as in the modal sequence) or prior to these more proximal elements (Fig. 3E and F). The timing of metatarsal III and IV ossification relative to the ischia is another source of variation in this analysis, in addition to the already mentioned first phalanges of the first digits. When excluding ambiguity, there are similar levels of variation as recovered in the forelimb and only slightly elevated levels of sequence variability.

## Discussion

### Variation and variability in the model species *M. domestica*

Our analysis revealed several important features of skeletal development in *M. domestica* and analyses of ontogenetic sequences more generally. The modal sequences of the whole skeleton and individual partition OSAs match the large-scale patterns of prior studies. For both forelimb and hindlimb partitions, the stylopodial and zeugopodial elements ossified first, followed by the most distal phalanges, then the metapodials and more proximal phalanges, and finally the carpals or tarsals. For the skull, the facial skeleton was the first to initiate ossification, followed closely by portions of the palate and skull base before the cranial vault elements, and finally more internal braincase and middle ear elements. However, in contrast to previously published ossification sequences for *M. domestica*, our study showed the precise timing of events were clearly polymorphic and no single sequence could result in the observed semaphoronts. These results reinforce other analyses that document the existence of variation in the timing and sequence of ossification within teleosts (Mabee et al. 2000; de Jong et al. 2009), birds (Mitgutsch et al. 2011), squamates (Maisano 2002), and mammals (Colbert and Rowe 2008; Wilson et al. 2010). The whole skeleton OSA recovered more than 5,000 ontogenetic sequences, which is a greater level of sequence polymorphism than recovered in prior studies using OSA to understand ontogeny in the tapir skull (Colbert 1999), human carpels (Colbert and Rowe 2008), and extinct species of theropod dinosaurs (Griffin 2018) and lepospondyl amphibians (Olori 2013), but less than extant cichlid fish (de Jong et al. 2009). Given the link between sample size and recovered variation (e.g., Colbert 1999; Visscher 2008; Garamszegi and Møller 2010), these data strongly suggest that studies not using OSA likely under-sample this natural variation in the sequence of ossification. Further, this raises questions about the meaning or utility of “typified” sequences, which attempt to find a “consensus” from such variation.

Our dataset includes multiple examples of specimens which possess equal maturity scores, but their individual event scores necessitate the existence of multiple ontogenetic patterns (e.g., the palatines and exoccipitals in TMM‐7619 and 7625; Fig. 6). Even if our analysis overestimates the true number of ontogenetic sequences, the occurrence of semaphoronts incompatible with a single sequence underscores the biological reality of such intraspecific variation in ontogeny. However, the observed variation in the onset of ossification was remarkably limited and does not render such studies intractable. Although there was significantly more variation recovered by OSA than other methods, these sequences represent less than 1% of the total theoretical fully‐resolved sequences (1.244 × 10^142^ for 92 events). Characterizing this squence polymorphism is critical to understanding which aspects of skeletal ontogeny are reliable indicators of maturity and, therefore, comparable across species and evolutionary transitions.

In addition to this sequence polymorphism, we also recovered substantial sequence variability across *M. domestica* ontogeny. The majority of specimens (74%) included in the whole skeleton OSA were reconstructed to have followed non-modal sequences and several alternative developmental patterns were nearly as common in the population as the modal sequences. Thus, the propensity to express variation—sequence variability *sensu*Wagner and Altenberg (1996)—in ossification sequence appears to be relatively high in *M. domestica*. Some of the variation probably reflects a lack of constraint among events or across modules in the whole skeleton OSA, as the precise timing of phalangeal ossification is unlikely to impact cranial ontogeny either structurally or functionally. The more precise timing (i.e., shorter sequence segments) and reduced levels of sequence polymorphism and variability observed in the forelimb and hindlimb portions are further evidence of this. The extreme level of sequence polymorphism in the skull alone, in contrast, suggests that this variability is a fundamental aspect of ontogeny. Without quantifying the propensity for variation, it might be assumed that the variation in ontogeny would be expressed in only a small portion of the population and therefore would not affect comparative analyses. Our results demonstrate that it should not be assumed *a priori* that variation and variability will not affect the reconstruction of developmental patterns. Our analysis of individual partitions recovered more variation and variability in early hindlimb ossification events than for the forelimb and cranial datasets (Fig. 4). Developmental constraints and integration have been proposed to explain reduced disparity in marsupial forelimb (Sears et al. 2012; Sears 2014; Kelly et al. 2019) and facial/oral skeletal anatomy (Bennet and Goswami 2013; Goswami et al. 2016; Spiekman and Werneburg 2017) relative to other mammalian clades. It is possible that this is reflected in our results and sequence variation and variability may themselves be modular in *M. domestica*. However, this variation is proportional to the inclusion of appropriately mature semaphoronts that could break up events early in ontogeny, which has been shown to affect our ability to understand patterns of variation (Colbert 1999; Hernandez et al. 2006; Kelley 2007; Colbert and Rowe 2008; Visscher 2008; Garamszegi and Møller 2010). Thus, future analyses are needed to assess whether variation in these early events reflects potential developmental constraints or sampling bias.

### Comparisons with published ossification sequences

The sequence of skeletal ossification exhibited by *M. domestica* has been previously described using traditional age arranging methods to reconstruct a single typical sequence for cranial (Clark and Smith 1993; Nunn and Smith 1998; Hautier et al. 2011) and appendicular subregions (Weisbecker et al. 2008). These prior studies present the reconstructed sequence of events as a list of elements and the day at which ossification was initiated, which we have translated into sequence position plots (treating events on the same day as unresolved) to enable comparisons with the results from OSA.

Although Clark and Smith (1993) studied all 25 cranial ossification events included in our analysis, subsequent studies focused on fewer elements (12 for Nunn and Smith 1998; 17 for Hautier et al. 2011). Although this discrepancy complicates comparisons, it is clear that when all three prior sequences are compared to the modal sequences recovered by OSA the majority of cranial events occur in a different sequence (13/25, 7/12, and 12/17). Compared with modal sequences from OSA, the Clark and Smith (1993) reconstruction (Fig. 5B) has earlier ossification of the lacrimals, vomer, nasals, orbitosphenoids, and mallei, while the initial ossification of the exoccipitals, jugals, basioccipital, alisphenoids, basisphenoid, presphenoids, incudes, and stapedes are delayed. Although the Nunn and Smith (1998) sequence matches the earlier study fairly well, distinct differences are clear: the frontals, squamosals, and parietals ossify earlier and the exoccipitals, basioccipital, alisphenoids, and jugals ossify later than our modal sequences (Fig. 5C). In contrast, the sequence presented by Hautier and colleagues (2011) is quite different. Relative to the modal sequences recovered by OSA, that study shows delayed ossification of the palatines, pterygoids, basisphenoid, and periotics and earlier ossification of the frontals, squamosals, jugals, lacrimals, parietals, nasals, alisphenoids, and orbitosphenoids (Fig. 5D). Importantly, these three prior analyses are in greater disagreement with each other, than any one is to the total variation recovered by OSA.

The forelimb and hindlimb ossification sequences reconstructed by Weisbecker and colleagues (2008) are less well resolved. This is partially an artifact of documenting only the day at which ossification begins, rather than scoring specimens for each ossification event—which has the effect of reducing each analysis to sampling only four unique semaphoronts. Further, all phalanges and metapodials were treated as a group that ossify simultaneously. However, Weisbecker and colleagues (2008) present no evidence for co-occurance of these events (instead of an unresolved sequence) and our results using OSA demonstrate conclusively these events are not simultaneous. Thus, in the forelimb (Fig. 7B), the clavicles were recognized as ossifying first. The rest of the pectoral girdle, arm, and manual phalanges ossify next, in an unresolved sequence, followed by the metacarpals and then finally all of the carpals. Only the clavicles were better resolved than in our modal sequence, but this timing still falls within the range recovered by OSA. In contrast, all other events were resolved more precisely by OSA. Further, in our study metacarpals II, III, and IV ossified much earlier, several phalanges ossified later, and the fourth distal carpals were resolved to a single position prior to all other carpals. For the hindlimb (Fig. 8B), the femora, tibiae, fibulae, ilia, and epipubes ossify in a single sequence segment prior to the ischia (the only individually resolved event). Subsequently all the pedal phalanges, metatarsals, and the pubes ossified in one unresolved sequence segment, followed finally by the ossification of the tarsals. The modal sequence in our study recovered similar patterns, except the ossification of epipubes and ischia occur earlier and distal phalanges of digits II through V ossify later in ontogeny. Importantly, these differences were only captured because our study included semaphoronts with event scores that were not sampled by prior studies. The disagreements across cranial and appendicular sequences are significant, however, when the full variation recovered by OSA is considered, it is clear that these discrepancies are not caused by comparing fundamentally incoherent datasets.

When non‐modal sequences are considered, the previously described cranial ossification sequences generally fall within the range of sequence polymorphism recovered by OSA (Fig. 5). Our analysis recovered slightly more precise sequence positions for earlier events (especially the palatines, prearticulars/gonials, frontals, and exoccipitals) than prior studies, but only a handful of events (vomer, ischia, and epipubes) fell outside of the range of sequence positions recovered by OSA. Although later events appear well‐resolved in prior studies, the sequence polymorphism found by OSA demonstrates that there is substantially greater variation. These comparisons further suggest the analysis by Hautier and colleagues (2011) sampled additional variation not found in our analysis, as they reconstructed the ossification of the pterygoids to their final three events but it occurred in the first 60% of all sequences recovered by OSA. Although the major differences in appendicular sequences were due to the lack of resolution, the early hindlimb ossification events, in particular, show more variability than previously recognized. Across all hindlimb sequences, only the ischia and epipubes were recovered in fundamentally later portions of ontogeny by OSA (Fig. 8). Although non‐modal forelimb sequences introduced minor discrepancies, in general the previously published sequences fell within the range of sequence polymorphism recovered by OSA (Fig. 7). Thus, these previously published ossification sequences are not “incorrect” but are rather a subset of the variation and variability within *Monodelphis*. The fact that our analysis was able to recover the sequences of alternative methods, in addition to sequences missed by other methods, is a testament to the robustness of OSA. This finding matches prior work showing OSA is superior to other methods of sequence reconstruction (de Jong et al. 2009; Olori 2013). Further, this demonstrates the importance of quantifying ontogenetic variation as there were more sequences recovered by OSA that were even more incongruent with the sequence described in previous studies, for the few that were congruent.

### Implications for comparative studies

The previously published sequences for *M. domestica* have been used in multiple comparative analyses characterizing the evolution of mammalian ontogeny (e.g., Sánchez‐Villagra et al. 2008; Weisbecker et al. 2008; Sears 2009; Hautier et al. 2011; Koyabu et al. 2011; Weisbecker 2011), which generally have found the ossification of the face and forelimbs to occur earlier in marsupials than placentals (Clark and Smith 1993; Weisbecker et al. 2008; Sears 2009; Koyabu et al. 2011). Although “advanced” forelimb development is likely ancestral for vertebrates, the initial embryonic differences found in marsupials are clearly more dramatic than the near synchrony observed in most amniotes (Bininda-Emonds et al. 2007). Historically, these developmental differences were thought to be related to selective pressure on marsupial neonates to reach their mother's pouch and suckle after birth (Lillegraven 1975; Renfree 1983). However, genetic explorations of limb development, in *M. domestica* specifically (Keyte and Smith 2010) and marsupials more broadly (Sears et al. 2012; Dowling et al. 2016), suggest the key developmental mechanism involved is actually a delay in hindlimb development rather than a changes to the forelimb. Specifically, a delay in *Fgf8* expression in the hindlimb but not the forelimb relative to other mammals has been identified as a putative mechanism for the evolution of this pattern (Keyte and Smith 2010). The genetics of precocial facial development are not as well understood, but geometric morphometric studies imply the facial skeleton is under strong constraints (Bennet and Goswami 2013; Goswami et al. 2016; Spiekman and Werneburg 2017) potentially due to paedomorphosis from the ancestral therian ontogenetic trajectory (White et al. 2023). However, the variation and variability recovered by OSA presents important implications for such comparative analyses of developmental sequences.

The modal sequences reconstructed by OSA for *M. domestica* show evidence of some aspects of these large-scale patterns, including the early ossification of the face and forelimb relative to the hindlimb (Fig. 3). Critically, however, when the full set of ontogenetic sequences are considered, we find the differences among regions are minor and may reflect sampling biases more than developmental constraints. The delay in hindlimb ossification recovered in our analysis was only by a single sequence segment and most forelimb events overlap with hindlimb events either in the same sequence or in alternative sequences (Fig. 2). Importantly, the onset of ossification occurs 4–5 days after the initiation of hindlimb outgrowth (Keyte and Smith 2010) and, therefore, may only partially reflect these initial developmental shifts rather than any specific evolution of ossification itself. Clearly there are important differences in the large-scale developmental patterns among extant therians, but by comparing only single sequences, future comparative analyses run the risk of inferring interspecific differences based on only a portion of the population of developmental patterns within individual species. This underscores that the primary data in developmental studies are specimens, not ontogenetic sequences, and each specimen needs to be considered individually. By scoring each specimen individually for all events and constructing specimen‐event and semaphoront‐event matrices, researchers can increase the repeatability and transparency of studies.

The significant variation recovered for *M. domestica* is clear evidence that sequence polymorphism and variability are inherent aspects of ontogeny that must be accounted for in comparative studies. Otherwise, the reconstructed differences and evolutionary patterns may be correct for only particular portions of the population. The range of variation and the frequency with which that variation is expressed (i.e., variability) in the population are crucial aspects of ontogeny as they are the source of variation upon which selection can act. Different species may have very similar modal sequences, but differ dramatically in their range of sequence polymorphism or sequence variability. Without quantifying and comparing this variation and variability, it will be impossible to identify the reliable markers of maturity or stable ontogenetic patterns. As one example, in both of our forelimb and hindlimb analyses, the carpals and tarsals never began to ossify until all other elements had begun to ossify. Moreover, this was not due to a lack of specimen data as several specimens from multiple ages represented the same semaphoront in which only the carpals or tarsals had yet ossified. This may reflect a genetic or mechanical constraint that prevents the ossification of carpals and tarsals until the end of limb development. By identifying where sequences diverge or converge and where sequence polymorphism is concentrated or not, OSA provides a unique window into potential developmental biases. By fully characterizing and comparing these aspects of ontogeny across species, in addition to the sequences, future studies will be better able to address the role ontogenetic variation and developmental constraint play in the evolution of developmental patterns.

## Conclusion

Our study has revealed previously unrecognized variation and variability in the skeletal development of *M. domestica*, an important model organism in evolutionary developmental biology. These patterns were only detectable because OSA allows such variation to be characterized and requires analyses to treat individual specimens, not typological sequences, as the primary data in developmental studies. The remarkable variation recovered during skeletal development in *M. domestica* demonstrates the importance of not only accounting for, but directly incorporating intraspecific ontogenetic variation into interspecific and evolutionary comparisons. This variation is a biological reality even if it complicates analyses. Our study recovered modal sequences with similarities to prior work, including earlier ossification of the facial skeleton and a slight offset between forelimb and hindlimb development. However, this represents only a small portion of the reconstructed ossification sequences. Our results demonstrate that interspecific comparisons that do not incorporate this variation likely reflect only a portion of the full range of sequence polymorphism within the population. This further emphasizes the importance of appropriate sample sizes and scoring individual specimens, as this is directly proportional to the reconstructed range of variation and resolution of ontogenetic sequences. Ontogenetic sequence analysis, like the phylogenetic frameworks it builds upon, requires that a species’ ontogeny be conceptualized not as a single pathway from embryo to fully mature adult, but instead as a network of individuals at specific instances along a multitude of ontogenetic pathways. This has the potential to revolutionize developmental studies just as the advances in phylogenetic methods have enabled biologist to grapple with complexity and uncertainty to more completely understand the fundamental processes generating evolutionary patterns. Future studies utilizing OSA to conduct comparative analyses within this framework will be able to address key questions about how ontogeny itself evolves and the role developmental variation plays in evolutionary transitions in the fossil record.

## Supplementary Material

obae024_Supplemental_File

## Data Availability

The data underlying this article are available in the article and its online supplementary material.
