# Supplementary material for: Variation and Variability in Skeletal Ossification of the Gray Short-tailed Opossum, Monodelphis domestica
Source: Integr Org Biol. 2024 Jul 3;6(1):obae024. doi: 10.1093/iob/obae024 (PMC11305135; doi:10.1093/iob/obae024)
Supplement: obae024_Supplemental_File [file obae024_supplemental_file.zip › IOB Monodelphis OSA Supplementary Materials.pdf]

Supplementary Materials

for

Variation and variability in skeletal ossification of the gray short-tailed opossum, *Monodelphis domestica*

Supplementary Methods 2

List of Developmental Events 2

Supplementary Tables 4

Table S1. Event Score Matrix 4

Supplementary Figures 13

Figure S1. Detailed whole skeleton OSA Network 13

Figure S2. Detailed cranial OSA Network 15

Figure S3. Detailed forelimb OSA Network 16

Figure S4. Detailed hindlimb OSA Network 17

Supplementary Methods

List of Developmental Events

The initiation of ossification (state “1") was assessed based on clear red staining or clear spicules on individual cleared-and-stained specimens. All bilaterally symmetric elements are listed as plural as in all specimens both sides were identical in our dataset.

Ev 1. The premaxillae are (0) not ossified or (1) ossified.
Ev 2. The maxillae are (0) not ossified or (1) ossified.
Ev 3. The palatines are (0) not ossified or (1) ossified.
Ev 4. The pterygoids are (0) not ossified or (1) ossified.
Ev 5. The dentaries are (0) not ossified or (1) ossified.
Ev 6. The squamosals are (0) not ossified or (1) ossified.
Ev 7. The vomer is (0) not ossified or (1) ossified.
Ev 8. The lacrimals are (0) not ossified or (1) ossified.
Ev 9. The ectotympanics are (0) not ossified or (1) ossified.
Ev 10. The prearticulars (gonials) are (0) not ossified or (1) ossified.
Ev 11. The nasals are (0) not ossified or (1) ossified.
Ev 12. The jugals are (0) not ossified or (1) ossified.
Ev 13. The parietals are (0) not ossified or (1) ossified.
Ev 14. The frontals are (0) not ossified or (1) ossified.
Ev 15. The interparietal are (0) not ossified or (1) ossified.
Ev 16. The exoccipitals are (0) not ossified or (1) ossified.
Ev 17. The basioccipital is (0) not ossified or (1) ossified.
Ev 18. The alisphenoids are (0) not ossified or (1) ossified.
Ev 19. The basisphenoid is (0) not ossified or (1) ossified.
Ev 20. The mallei are (0) not ossified or (1) ossified.
Ev 21. The periotics are (0) not ossified or (1) ossified.
Ev 22. The presphenoids are (0) not ossified or (1) ossified.
Ev 23. The orbitosphenoids are (0) not ossified or (1) ossified.
Ev 24. The incudes are (0) not ossified or (1) ossified.
Ev 25. The stapes are (0) not ossified or (1) ossified.
Ev 26. The scapulae are (0) not ossified or (1) ossified.
Ev 27. The clavicles are (0) not ossified or (1) ossified.
Ev 28. The humeri are (0) not ossified or (1) ossified.
Ev 29. The radii are (0) not ossified or (1) ossified.
Ev 30. The ulnae are (0) not ossified or (1) ossified.
Ev 31. The radialia (0) not ossified or (1) ossified.
Ev 32. The intermedia of the carpus are (0) not ossified or (1) ossified.
Ev 33. The ulnares are (0) not ossified or (1) ossified.
Ev 34. The pisiforms of the carpus are (0) not ossified or (1) ossified.
Ev 35. The centralia of the carpus are (0) not ossified or (1) ossified.
Ev 36. The first (1st) distal carpals are (0) not ossified or (1) ossified.

Ev 37. The second (2nd) distal carpals are (0) not ossified or (1) ossified.
Ev 38. The third (3rd) distal carpals are (0) not ossified or (1) ossified.
Ev 39. The fourth (4th) distal carpals are (0) not ossified or (1) ossified.
Ev 40. The first (1st) metacarpals are (0) not ossified or (1) ossified.
Ev 41. The second (2nd) metacarpals are (0) not ossified or (1) ossified.
Ev 42. The third (3rd) metacarpals are (0) not ossified or (1) ossified.
Ev 43. The fourth (4th) metacarpals are (0) not ossified or (1) ossified.
Ev 44. The fifth (5th) metacarpals are (0) not ossified or (1) ossified.
Ev 45. The first (1st) phalanx of the first (1st) manual digits are (0) not ossified or (1) ossified.
Ev 46. The second (2nd) phalanx of the first (1st) manual digits are (0) not ossified or (1) ossified.
Ev 47. The first (1st) phalanx of the second (2nd) manual digits are (0) not ossified or (1) ossified.
Ev 48. The second (2nd) phalanx of second (2nd) manual digits are (0) not ossified or (1) ossified.
Ev 49. The third (3rd) phalanx of the second (2nd) manual digits are (0) not ossified or (1) ossified.
Ev 50. The first (1st) phalanx of the third (3rd) manual digits are (0) not ossified or (1) ossified.
Ev 51. The second (2nd) phalanx of the third (3rd) manual digits are (0) not ossified or (1) ossified.
Ev 52. The third (3rd) phalanx of the third (3rd) manual digits are (0) not ossified or (1) ossified.
Ev 53. The first (1st) phalanx of the fourth (4th) manual digits are (0) not ossified or (1) ossified.
Ev 54. The second (2nd) phalanx of the fourth (4th) manual digits are (0) not ossified or (1) ossified.
Ev 55. The third (3rd) phalanx of the fourth (4th) manual digits are (0) not ossified or (1) ossified.
Ev 56. The first (1st) phalanx of the fifth (5th) manual digits are (0) not ossified or (1) ossified.
Ev 57. The second (2nd) phalanx of the fifth (5th) manual digits are (0) not ossified or (1) ossified.
Ev 58. The third (3rd) phalanx of the fifth (5th) manual digits are (0) not ossified or (1) ossified.
Ev 59. The ilia are (0) not ossified or (1) ossified.
Ev 60. The ischia are (0) not ossified or (1) ossified.
Ev 61. The pubes are (0) not ossified or (1) ossified.
Ev 62. The epipubes are (0) not ossified or (1) ossified.
Ev 63. The femora are (0) not ossified or (1) ossified.
Ev 64. The tibiae are (0) not ossified or (1) ossified.
Ev 65. The fibulae are (0) not ossified or (1) ossified.
Ev 66. The tibialia are (0) not ossified or (1) ossified.
Ev 67. The intermedia of the tarsi are (0) not ossified or (1) ossified.
Ev 68. The fibularia are (0) not ossified or (1) ossified.
Ev 69. The centralia of the carpi are (0) not ossified or (1) ossified.
Ev 70. The first (1st) distal tarsals are (0) not ossified or (1) ossified.
Ev 71. The second (2nd) distal tarsals are (0) not ossified or (1) ossified.
Ev 72. The third (3rd) distal tarsals are (0) not ossified or (1) ossified.
Ev 73. The fourth (4th) distal tarsals are (0) not ossified or (1) ossified.
Ev 74. The first (1st) metatarsals are (0) not ossified or (1) ossified.
Ev 75. The second (2nd) metatarsals are (0) not ossified or (1) ossified.
Ev 76. The third (3rd) metatarsals are (0) not ossified or (1) ossified.
Ev 77. The fourth (4th) metatarsals are (0) not ossified or (1) ossified.

Ev 78. The fifth (5th) metatarsals are (0) not ossified or (1) ossified.
Ev 79. The first (1st) phalanx of the first (1st) pedal digits are (0) not ossified or (1) ossified.
Ev 80. The second (2nd) phalanx of the first (1st) pedal digits are (0) not ossified or (1) ossified.
Ev 81. The first (1st) phalanx of the second (2nd) pedal digits are (0) not ossified or (1) ossified.
Ev 82. The second (2nd) phalanx of the second (2nd) pedal digits are (0) not ossified or (1) ossified.
Ev 83. The third (3rd) phalanx of the second (2nd) pedal digits are (0) not ossified or (1) ossified.
Ev 84. The first (1st) phalanx of the third (3rd) pedal digits are (0) not ossified or (1) ossified.
Ev 85. The second (2nd) phalanx of the third (3rd) pedal digits are (0) not ossified or (1) ossified.
Ev 86. The third (3rd) phalanx of the third (3rd) pedal digits are (0) not ossified or (1) ossified.
Ev 87. The first (1st) phalanx of the fourth (4th) pedal digits are (0) not ossified or (1) ossified.
Ev 88. The second (2nd) phalanx of the fourth (4th) pedal digits are (0) not ossified or (1) ossified.
Ev 89. The third (3rd) phalanx of the fourth (4th) pedal digits are (0) not ossified or (1) ossified.
Ev 90. The first (1st) phalanx of the fifth (5th) pedal digits are (0) not ossified or (1) ossified.
Ev 91. The second (2nd) phalanx of the fifth (5th) pedal digits are (0) not ossified or (1) ossified.
Ev 92. The third (3rd) phalanx of the fifth (5th) pedal digits are (0) not ossified or (1) ossified.

Supplementary Tables

Table S1. Event Score Matrix

This matrix contains the event scores for each specimen as scored for the whole skeleton dataset. Event numbers correspond to the event list (above) with "0" signifying no indication of ossified tissue, "1" signifying that the specimen had an ossified element, and "?" signifying that it was unknown or unclear whether or not ossification had occurred. This matrix was divided for cranial (events 1 — 25), forelimb (events 26 — 58), and hindlimb (events 59— 92) sub-region analyses. The analyzed nexus files can be found in a zip file in the associated online supplementary materials.

	Event Number																								
**Specimen ID**	**1**	**2**	**3**	**4**	**5**	**6**	**7**	**8**	**9**	**10**	**11**	**12**	**13**	**14**	**15**	**16**	**17**	**18**	**19**	**20**	**21**	**22**	**23**	**24**	**25**
TMM-7612 (0b)	0	0	0	0	0	0	0	0	0	0	0	0	0	0	0	0	0	0	0	0	0	0	0	0	0
TMM-7615 (1a)	0	0	0	0	0	0	0	0	0	0	0	0	0	0	0	0	0	0	0	0	0	0	0	0	0
TMM-7616 (1b)	1	1	1	0	1	0	0	0	0	0	0	0	0	0	0	1	0	0	0	0	0	0	0	0	0
TMM-7619 (1d)	1	1	1	0	1	0	0	0	0	0	0	0	0	0	0	0	0	0	0	0	0	0	0	0	0
TMM-7623 (2c)	1	1	1	0	1	0	0	0	0	0	0	0	0	0	0	1	0	0	0	0	0	0	0	0	0
TMM-7624 (2d)	1	1	1	1	1	0	0	1	1	1	1	1	1	1	1	1	0	0	0	0	0	0	0	0	0
TMM-7625 (3a)	1	1	0	0	1	0	0	0	?	?	0	0	0	0	0	1	0	0	0	0	0	0	0	0	0
TMM-7626 (3b)	1	1	1	1	1	1	1	1	1	1	0	1	1	1	1	1	1	1	1	0	0	0	0	0	0
TMM-7627 (3c)	1	1	1	1	1	1	1	1	1	1	1	1	?	1	?	1	0	0	0	0	0	0	0	0	0
TMM-7628 (day 3)	1	1	1	1	1	0	0	0	0	1	0	0	1	1	1	1	0	0	0	0	0	0	0	0	0
TMM-7629 (4a)	1	1	1	1	1	0	0	0	1	1	0	1	0	1	0	1	1	0	0	0	0	0	0	0	0
TMM-7633 (5b)	1	1	1	1	1	1	0	0	1	1	1	1	1	1	1	1	0	0	0	0	0	0	0	0	0
TMM-7635 (day 6)	1	1	1	0	1	0	0	1	0	1	1	0	0	1	1	1	1	0	1	0	0	0	0	0	0
TMM-7636 (7a)	1	1	1	1	1	1	1	1	1	1	1	1	0	1	1	1	1	1	1	0	0	0	0	0	0
TMM-7637 (7b)	1	1	1	1	1	1	0	1	1	1	0	1	0	1	0	1	1	1	1	0	0	0	0	0	0
TMM-7638 (7c)	1	1	1	1	1	1	1	0	1	1	1	1	1	1	1	1	1	1	1	0	0	0	1	0	0
TMM-7639 (day 7)	1	1	1	1	1	1	1	1	1	1	1	1	0	1	1	1	1	1	1	0	0	0	0	0	0
TMM-7640 (8a)	1	1	1	1	1	1	?	1	1	1	1	1	1	1	1	1	1	1	1	0	0	0	1	0	0
TMM-7641 (8b)	1	1	1	1	1	1	1	1	1	1	1	1	1	1	1	1	1	1	1	0	0	0	0	0	0

	Event Number																								
**Specimen ID**	**1**	**2**	**3**	**4**	**5**	**6**	**7**	**8**	**9**	**10**	**11**	**12**	**13**	**14**	**15**	**16**	**17**	**18**	**19**	**20**	**21**	**22**	**23**	**24**	**25**
TMM-7642 (8c)	1	1	1	1	1	1	1	1	1	1	1	1	1	1	1	1	1	1	1	0	0	0	0	0	0
TMM-7643 (day 8)	1	1	1	1	1	1	1	1	1	1	1	1	1	1	1	1	1	1	1	0	1	0	0	0	0
TMM-7644 (9a1)	1	1	1	1	1	1	1	1	1	1	1	1	1	1	1	1	1	1	1	0	0	0	0	?	?
TMM-7645 (9a2)	1	1	1	1	1	1	1	1	1	1	1	1	1	1	1	1	1	1	1	0	0	0	0	0	0
TMM-7646 (9b)	1	1	1	1	1	1	1	1	1	1	1	1	1	1	1	1	1	1	1	0	0	0	0	0	0
TMM-7647 (10a)	1	1	1	1	1	1	1	1	1	1	1	1	1	1	1	1	1	1	1	1	0	1	0	0	0
TMM-7649 (11a)	1	1	1	1	1	1	1	0	1	1	1	1	1	1	1	1	1	1	1	0	0	1	0	0	0
TMM-7650 (11b)	1	1	1	1	1	1	1	1	1	1	1	1	1	1	1	1	1	1	1	0	0	1	0	0	0
TMM-7654 (13a)	1	1	1	1	1	1	1	1	1	1	1	1	1	1	1	1	1	1	1	1	1	1	0	0	0
TMM-7659 (15a)	1	1	1	1	1	1	1	1	1	1	1	1	1	1	1	1	1	1	1	1	1	1	0	1	1
TMM-7660 (15b)	1	1	1	1	1	1	1	1	1	1	1	1	1	1	0	1	1	1	1	1	1	1	0	0	1
TMM-7662 (15d)	1	1	1	1	1	1	1	1	1	1	1	1	1	1	1	1	1	1	1	1	1	1	0	1	0
TMM-7663 (15e)	1	1	1	1	1	1	1	1	1	1	1	1	1	1	1	1	1	1	1	1	1	1	0	0	0
TMM-7664 (15f)	1	1	1	1	1	1	1	1	1	1	1	1	1	1	1	1	1	1	1	1	1	1	0	0	0
TMM-7665 (15g1)	1	1	1	1	1	1	1	1	1	1	1	1	1	1	1	1	1	1	1	1	1	1	0	0	0
TMM-7667 (16a)	1	1	1	1	1	1	1	1	1	1	1	1	1	1	1	1	1	1	1	1	1	1	0	0	0
TMM-7671 (17b)	1	1	1	1	1	1	1	1	1	1	1	1	1	1	1	1	1	1	1	1	1	1	0	1	0
TMM-7679 (20a)	1	1	1	1	1	1	1	1	1	1	1	1	1	1	1	1	1	1	1	1	1	1	1	1	1
TMM-7684 (21b)	1	1	1	1	1	1	1	1	1	1	1	1	1	1	1	1	1	1	1	1	1	1	?	1	1

	Event Number																								
**Specimen ID**	**26**	**27**	**28**	**29**	**30**	**31**	**32**	**33**	**34**	**35**	**36**	**37**	**38**	**39**	**40**	**41**	**42**	**43**	**44**	**45**	**46**	**47**	**48**	**49**	**50**
TMM-7612 (0b)	0	0	0	0	0	0	0	0	0	0	0	0	0	0	0	0	0	0	0	0	0	0	0	0	0
TMM-7615 (1a)	0	0	0	0	0	0	0	0	0	0	0	0	0	0	0	0	0	0	0	0	0	0	0	0	0
TMM-7616 (1b)	1	1	1	1	1	0	0	0	0	0	0	0	0	0	0	0	0	0	0	0	1	0	0	1	0
TMM-7619 (1d)	1	1	1	1	1	0	0	0	0	0	0	0	0	0	0	0	0	0	0	0	1	0	0	1	0
TMM-7623 (2c)	1	1	1	1	1	0	0	0	0	0	0	0	0	0	0	0	0	0	0	0	1	0	0	1	0
TMM-7624 (2d)	1	1	1	1	1	0	0	0	0	0	0	0	0	0	0	0	0	0	0	0	1	0	0	1	0
TMM-7625 (3a)	1	1	1	1	1	0	0	0	0	0	0	0	0	0	0	0	0	0	0	0	1	0	0	1	0
TMM-7626 (3b)	1	1	1	1	1	0	0	0	0	0	0	0	0	0	0	0	0	0	0	0	1	0	0	1	0
TMM-7627 (3c)	1	1	1	1	1	0	0	0	0	0	0	0	0	0	0	0	0	0	0	0	1	0	0	1	0
TMM-7628 (day 3)	1	1	1	1	1	0	0	0	0	0	0	0	0	0	0	0	0	0	0	0	1	0	0	1	0
TMM-7629 (4a)	1	1	1	1	1	0	0	0	0	0	0	0	0	0	0	0	0	0	0	0	1	0	0	1	0
TMM-7633 (5b)	1	1	1	1	1	0	0	0	0	0	0	0	0	0	0	1	1	1	0	0	1	0	0	1	1
TMM-7635 (day 6)	1	1	1	1	1	0	0	0	0	0	0	0	0	0	0	0	0	0	0	0	0	0	0	0	0
TMM-7636 (7a)	1	1	1	1	1	0	0	0	0	0	0	0	0	0	0	1	1	1	0	1	1	1	1	1	1
TMM-7637 (7b)	1	1	1	1	1	0	0	0	0	0	0	0	0	0	0	1	1	1	0	0	1	1	1	1	1
TMM-7638 (7c)	1	1	1	1	1	0	0	0	0	0	0	0	0	0	0	1	1	1	0	1	1	1	1	1	1
TMM-7639 (day 7)	1	1	1	1	1	0	0	0	0	0	0	0	0	0	0	1	1	1	0	0	1	0	0	1	0
TMM-7640 (8a)	1	1	1	1	1	0	0	0	0	0	0	0	0	0	0	1	1	1	0	0	1	1	1	1	1
TMM-7641 (8b)	1	1	1	1	1	0	0	0	0	0	0	0	0	0	0	1	1	1	0	1	1	1	1	1	1

	Event Number																								
**Specimen ID**	**26**	**27**	**28**	**29**	**30**	**31**	**32**	**33**	**34**	**35**	**36**	**37**	**38**	**39**	**40**	**41**	**42**	**43**	**44**	**45**	**46**	**47**	**48**	**49**	**50**
TMM-7642 (8c)	1	1	1	1	1	0	0	0	0	0	0	0	0	0	0	1	1	1	0	0	1	0	0	1	0
TMM-7643 (day 8)	1	1	1	1	1	0	0	0	0	0	0	0	0	0	0	1	1	1	0	0	1	1	1	1	1
TMM-7644 (9a1)	1	1	1	1	1	0	0	0	0	0	0	0	0	0	1	1	1	1	1	1	1	1	1	1	1
TMM-7645 (9a2)	1	1	1	1	1	0	0	0	0	0	0	0	0	0	1	1	1	1	1	1	1	1	1	1	1
TMM-7646 (9b)	1	1	1	1	1	0	0	0	0	0	0	0	0	0	1	1	1	1	1	1	1	1	1	1	1
TMM-7647 (10a)	1	1	1	1	1	0	0	0	0	0	0	0	0	0	1	1	1	1	1	1	1	1	1	1	1
TMM-7649 (11a)	1	1	1	1	1	0	0	0	0	0	0	0	0	0	1	1	1	1	1	1	1	1	1	1	1
TMM-7650 (11b)	1	1	1	1	1	0	0	0	0	0	0	0	0	0	1	1	1	1	1	1	1	1	1	1	1
TMM-7654 (13a)	1	1	1	1	1	0	0	0	0	0	0	0	0	0	1	1	1	1	1	1	1	1	1	1	1
TMM-7659 (15a)	1	1	1	1	1	0	0	0	0	0	0	0	0	0	1	1	1	1	1	1	1	1	1	1	1
TMM-7660 (15b)	1	1	1	1	1	0	0	0	0	0	0	0	0	0	1	1	1	1	1	1	1	1	1	1	1
TMM-7662 (15d)	1	1	1	1	1	0	0	0	0	0	0	0	0	0	1	1	1	1	1	1	1	1	1	1	1
TMM-7663 (15e)	1	1	1	1	1	0	0	0	0	0	0	0	0	0	1	1	1	1	1	1	1	1	1	1	1
TMM-7664 (15f)	1	1	1	1	1	0	0	0	0	0	0	0	0	0	1	1	1	1	1	1	1	1	1	1	1
TMM-7665 (15g1)	1	1	1	1	1	0	0	0	0	0	0	0	0	0	1	1	1	1	1	1	1	1	1	1	1
TMM-7667 (16a)	1	1	1	1	1	0	0	0	0	0	0	0	0	0	1	1	1	1	1	1	1	1	1	1	1
TMM-7671 (17b)	1	1	1	1	1	0	0	0	0	0	0	0	0	0	1	1	1	1	1	1	1	1	1	1	1
TMM-7679 (20a)	1	1	1	1	1	0	0	0	0	0	0	0	0	1	1	1	1	1	1	1	1	1	1	1	1
TMM-7684 (21b)	1	1	1	1	1	0	0	0	0	0	0	0	0	1	1	1	1	1	1	1	1	1	1	1	1

	Event Number																								
**Specimen ID**	**51**	**52**	**53**	**54**	**55**	**56**	**57**	**58**	**59**	**60**	**61**	**62**	**63**	**64**	**65**	**66**	**67**	**68**	**69**	**70**	**71**	**72**	**73**	**74**	**75**
TMM-7612 (0b)	0	0	0	0	0	0	0	0	0	0	0	0	0	0	0	0	0	0	0	0	0	0	0	0	0
TMM-7615 (1a)	0	0	0	0	0	0	0	0	0	0	0	0	0	0	0	0	0	0	0	0	0	0	0	0	0
TMM-7616 (1b)	0	1	0	0	1	0	0	1	0	0	0	0	0	0	0	0	0	0	0	0	0	0	0	0	0
TMM-7619 (1d)	0	1	0	0	1	0	0	1	0	0	0	0	0	0	0	0	0	0	0	0	0	0	0	0	0
TMM-7623 (2c)	0	1	0	0	1	0	0	1	0	0	0	0	0	0	0	0	0	0	0	0	0	0	0	0	0
TMM-7624 (2d)	0	1	0	0	1	0	0	1	0	0	0	0	0	0	0	0	0	0	0	0	0	0	0	0	0
TMM-7625 (3a)	0	1	0	0	1	0	0	1	0	0	0	0	0	0	0	0	0	0	0	0	0	0	0	0	0
TMM-7626 (3b)	0	1	0	0	1	0	0	1	?	?	?	?	?	?	?	0	0	0	0	0	0	0	0	0	0
TMM-7627 (3c)	0	1	0	0	1	0	0	1	1	0	0	0	1	1	1	0	0	0	0	0	0	0	0	0	0
TMM-7628 (day 3)	0	1	0	0	1	0	0	1	0	0	0	0	0	0	0	0	0	0	0	0	0	0	0	0	0
TMM-7629 (4a)	0	1	0	0	1	0	0	1	0	0	0	0	1	1	1	0	0	0	0	0	0	0	0	0	0
TMM-7633 (5b)	1	1	1	0	1	0	0	1	1	0	0	0	1	1	1	0	0	0	0	0	0	0	0	0	0
TMM-7635 (day 6)	0	0	0	0	0	0	0	0	0	0	0	0	1	1	?	?	?	?	?	?	?	?	?	?	?
TMM-7636 (7a)	1	1	1	1	1	0	0	0	1	0	0	0	1	1	1	0	0	0	0	0	0	0	0	0	0
TMM-7637 (7b)	1	1	1	1	1	0	0	1	1	0	0	0	1	1	1	0	0	0	0	0	0	0	0	0	0
TMM-7638 (7c)	1	1	1	1	1	0	0	1	1	0	0	0	1	1	1	0	0	0	0	0	0	0	0	0	0
TMM-7639 (day 7)	0	1	0	0	1	0	0	1	1	0	0	0	1	1	1	0	0	0	0	0	0	0	0	0	0
TMM-7640 (8a)	1	1	1	1	1	0	0	1	1	0	0	0	1	1	1	0	0	0	0	0	0	0	0	0	0
TMM-7641 (8b)	1	1	1	1	1	0	0	1	1	0	0	0	1	1	1	0	0	0	0	0	0	0	0	0	0

Specimen ID	Event Number 51	52	53	54	55	56	57	58	59	60	61	62	63	64	65	66	67	68	69	70	71	72	73	74	75
TMM-7642 (8c)	0	1	0	0	1	0	0	1	1	0	0	0	1	1	1	0	0	0	0	0	0	0	0	0	0
TMM-7643 (day 8)	1	1	1	1	1	0	1	1	1	0	0	0	1	1	1	0	0	0	0	0	0	0	0	0	0
TMM-7644 (9a1)	1	1	1	1	1	1	1	1	1	1	0	0	1	1	1	0	0	0	0	0	0	0	0	0	0
TMM-7645 (9a2)	1	1	1	1	1	0	0	1	1	0	0	0	1	1	1	0	0	0	0	0	0	0	0	0	0
TMM-7646 (9b)	1	1	1	1	1	1	1	1	1	1	0	0	1	1	1	0	0	0	0	0	0	0	0	0	0
TMM-7647 (10a)	1	1	1	1	1	1	1	1	1	1	0	0	1	1	1	0	0	0	0	0	0	0	0	0	0
TMM-7649 (11a)	1	1	1	1	1	1	1	1	1	1	0	0	1	1	1	0	0	0	0	0	0	0	0	0	0
TMM-7650 (11b)	1	1	1	1	1	1	1	1	1	1	0	0	1	1	1	0	0	0	0	0	0	0	0	0	0
TMM-7654 (13a)	1	1	1	1	1	1	1	1	1	1	1	1	1	1	1	0	0	0	0	0	0	0	0	1	1
TMM-7659 (15a)	1	1	1	1	1	1	1	1	1	1	1	1	1	1	1	0	0	0	0	0	0	0	0	1	1
TMM-7660 (15b)	1	1	1	1	1	1	1	1	1	1	1	1	1	1	1	0	0	0	0	0	0	0	0	1	1
TMM-7662 (15d)	1	1	1	1	1	1	1	1	1	1	1	1	1	1	1	0	0	0	0	0	0	0	0	1	1
TMM-7663 (15e)	1	1	1	1	1	1	1	1	1	1	1	1	1	1	1	0	0	0	0	0	0	0	0	0	1
TMM-7664 (15f)	1	1	1	1	1	1	1	1	1	1	1	1	1	1	1	0	0	0	0	0	0	0	0	1	1
TMM-7665 (15g1)	1	1	1	1	1	1	1	1	1	1	1	1	1	1	1	0	0	0	0	0	0	0	0	1	1
TMM-7667 (16a)	1	1	1	1	1	1	1	1	1	1	1	1	1	1	1	0	0	0	0	0	0	0	0	1	1
TMM-7671 (17b)	1	1	1	1	1	1	1	1	1	1	1	1	1	1	1	0	0	0	0	0	0	0	0	1	1
TMM-7679 (20a)	1	1	1	1	1	1	1	1	1	1	1	1	1	1	1	0	0	0	0	0	0	0	0	1	1
TMM-7684 (21b)	1	1	1	1	1	1	1	1	1	1	1	1	1	1	1	0	0	1	0	0	0	0	0	1	1

Specimen ID	Event Number 76	77	78	79	80	81	82	83	84	85	86	87	88	89	90	91	92
TMM-7612 (0b)	0	0	0	0	0	0	0	0	0	0	0	0	0	0	0	0	0
TMM-7615 (1a)	0	0	0	0	0	0	0	0	0	0	0	0	0	0	0	0	0
TMM-7616 (1b)	0	0	0	0	0	0	0	0	0	0	0	0	0	0	0	0	0
TMM-7619 (1d)	0	0	0	0	0	0	0	0	0	0	0	0	0	0	0	0	0
TMM-7623 (2c)	0	0	0	0	0	0	0	0	0	0	0	0	0	0	0	0	0
TMM-7624 (2d)	0	0	0	0	0	0	0	0	0	0	0	0	0	0	0	0	0
TMM-7625 (3a)	0	0	0	0	0	0	0	0	0	0	0	0	0	0	0	0	0
TMM-7626 (3b)	0	0	0	0	0	0	0	0	0	0	0	0	0	0	0	0	0
TMM-7627 (3c)	0	0	0	0	1	0	0	1	0	0	1	0	0	1	0	0	1
TMM-7628 (day 3)	0	0	0	0	1	0	0	1	0	0	1	0	0	1	0	0	1
TMM-7629 (4a)	0	0	0	0	0	0	0	0	0	0	0	0	0	0	0	0	0
TMM-7633 (5b)	0	0	0	0	0	0	0	0	0	0	0	0	0	0	0	0	0
TMM-7635 (day 6)	?	?	?	?	?	?	?	?	?	?	?	?	?	?	?	?	?
TMM-7636 (7a)	0	0	0	0	0	0	0	0	0	0	0	0	0	0	0	0	0
TMM-7637 (7b)	0	0	0	0	0	0	0	1	0	0	1	0	0	1	0	0	1
TMM-7638 (7c)	0	0	0	0	1	0	0	1	0	0	1	0	0	1	0	0	1
TMM-7639 (day 7)	0	0	0	0	0	0	0	0	0	0	0	0	0	0	0	0	0
TMM-7640 (8a)	0	0	0	0	1	0	0	1	0	0	1	0	0	1	0	0	1
TMM-7641 (8b)	0	0	0	0	0	0	0	1	0	0	1	0	0	1	0	0	1

	Event Number																
**Specimen ID**	**76**	**77**	**78**	**79**	**80**	**81**	**82**	**83**	**84**	**85**	**86**	**87**	**88**	**89**	**90**	**91**	**92**
TMM-7642 (8c)	1	1	0	0	1	0	0	1	0	0	1	0	0	1	0	0	1
TMM-7643 (day 8)	0	0	0	0	1	0	0	1	0	0	1	0	0	1	0	0	1
TMM-7644 (9a1)	0	0	0	0	1	0	0	1	0	0	1	0	0	1	0	0	1
TMM-7645 (9a2)	0	0	0	0	1	0	0	1	0	0	1	0	0	1	0	0	1
TMM-7646 (9b)	0	0	0	0	1	0	0	1	0	0	1	0	0	1	0	0	1
TMM-7647 (10a)	0	0	0	0	1	0	0	1	0	0	1	0	0	1	0	0	1
TMM-7649 (11a)	0	0	0	0	?	0	0	1	0	0	1	0	0	1	0	0	1
TMM-7650 (11b)	0	0	0	0	1	0	0	1	0	0	1	0	0	1	0	0	1
TMM-7654 (13a)	1	1	1	1	1	0	0	1	0	0	1	0	0	1	0	1	1
TMM-7659 (15a)	1	1	1	1	1	1	1	1	1	1	1	1	1	1	1	1	1
TMM-7660 (15b)	1	1	1	1	1	1	1	1	1	1	1	1	1	1	1	1	1
TMM-7662 (15d)	1	1	1	1	1	1	1	1	1	1	1	1	1	1	1	1	1
TMM-7663 (15e)	1	1	1	?	?	0	0	1	0	0	1	0	0	1	0	0	1
TMM-7664 (15f)	1	1	1	0	1	0	1	1	0	1	1	0	1	1	0	1	1
TMM-7665 (15g1)	1	1	1	1	1	1	1	1	1	1	1	1	1	1	1	1	1
TMM-7667 (16a)	1	1	1	1	1	1	1	1	1	1	1	1	1	1	1	1	1
TMM-7671 (17b)	1	1	1	1	1	1	1	1	1	1	1	1	1	1	1	1	1
TMM-7679 (20a)	1	1	1	1	1	1	1	1	1	1	1	1	1	1	1	1	1
TMM-7684 (21b)	1	1	1	1	1	1	1	1	1	1	1	1	1	1	1	1	1

Supplementary Figures

Figure S1. Detailed whole skeleton OSA Network

Detailed version of whole skeleton OSA network (Figure 1) labeled with semaphoronts and events. Observed semaphoronts are represented by open ellipses, with width proportional to frequency support, with unobserved semaphoronts reconstructed by OSA are represented by filled nodes. The modal sequences are shown by bold segments. Ambiguity resulted in semaphoronts with multiple possible maturities represented by double-dashed lines.

Detailed OSA of Skeletal Ossification

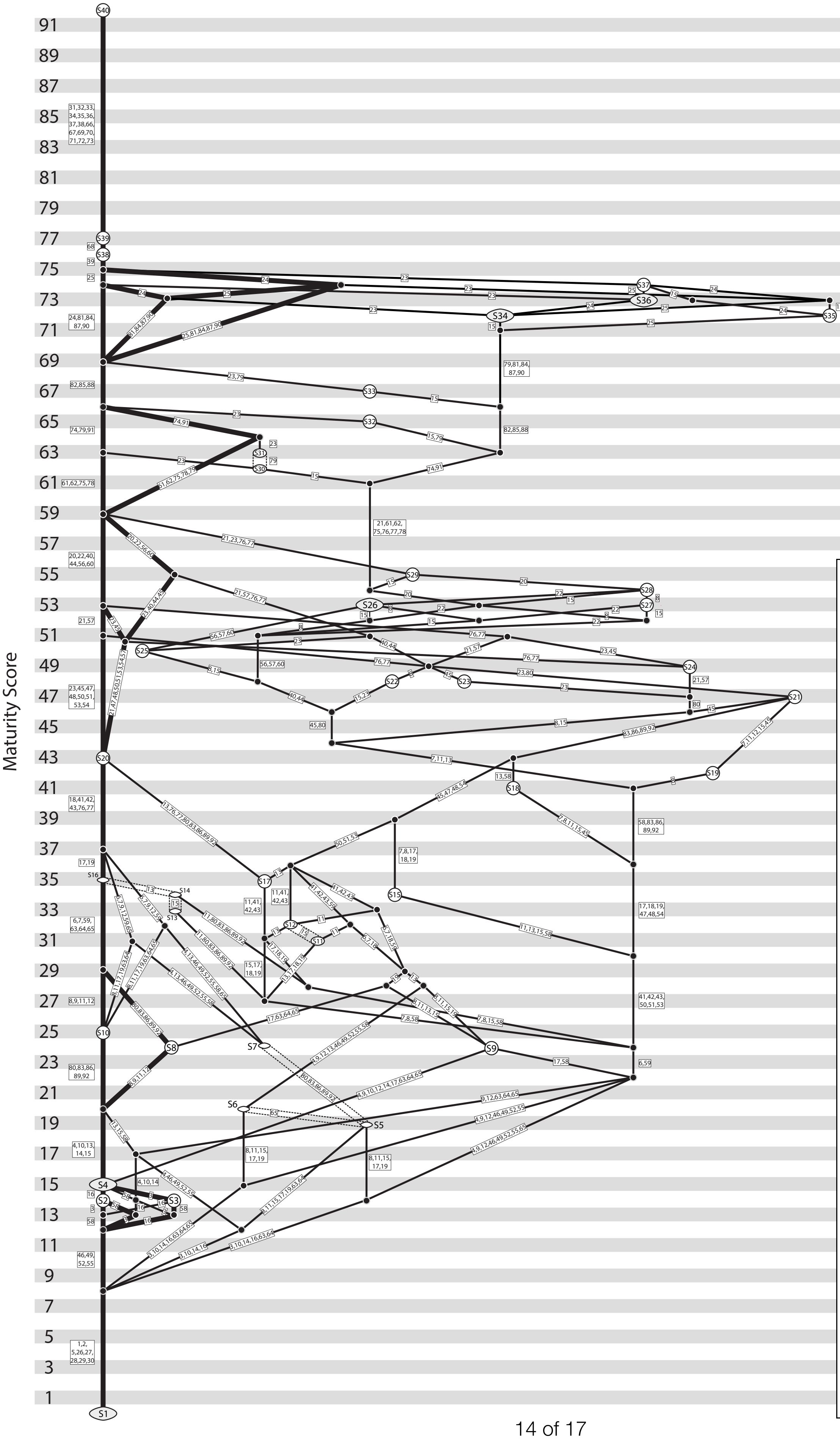

Semaphoront	Frequency Support	Specimen IDs
S40	n/a	hypothetical fully mature specimen
S39	1	21b
S38	1	20a
S37	1	15a
S36	2	15d, 17b
S35	1	15b
S34	2	15g1, 16a
S33	1	15f
S32	1	13a
S31	0.5	15e
S30	0.5	15e
S29	1	10a
S28	1	11b
S27	1	11a
S26	2	9a1, 9b
S25	1	9a2
S24	1	day8
S23	1	8a
S22	1	7c
S21	1	8b
S20	1	8c
S19	1	7b
S18	1	7a
S17	1	day7
S16	0.3333	3b
S15	1	5b
S14	0.3333	3b
S13	0.3333	3b
S12	0.5	3c
S11	0.5	3c
S10	1	day3
S9	1	4a
S8	1	2d
S7	0.3333	day6
S6	0.3333	day6
S5	0.3333	day6
S4	2	1b, 2c
S3	1	3a
S2	1	1d
S1	2	0b, 1a

Figure S2. Detailed cranial OSA Network

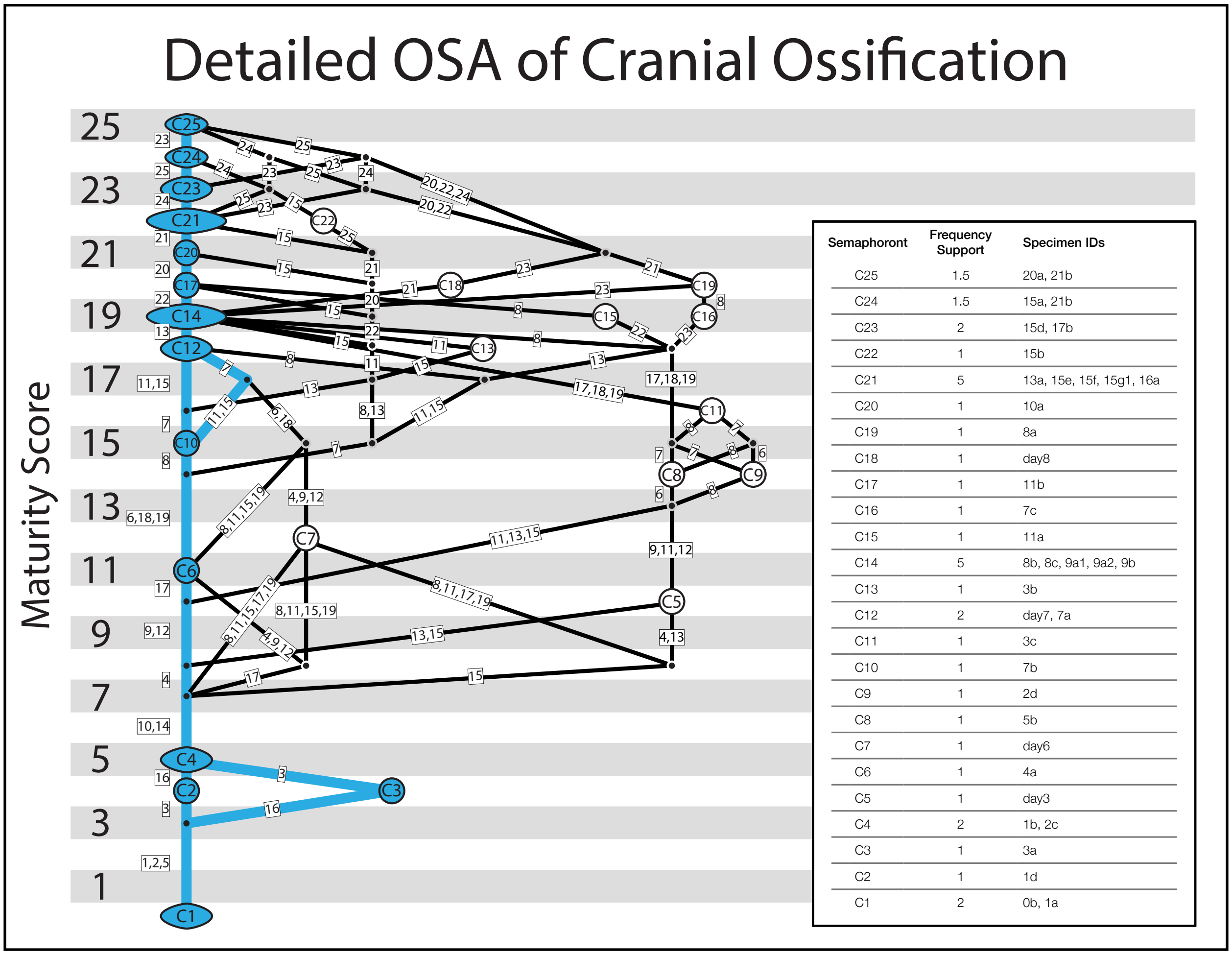

Semaphoront	Frequency Support	Specimen IDs
C25	1.5	20a, 21b
C24	1.5	15a, 21b
C23	2	15d, 17b
C22	1	15b
C21	5	13a, 15e, 15f, 15g1, 16a
C20	1	10a
C19	1	8a
C18	1	day8
C17	1	11b
C16	1	7c
C15	1	11a
C14	5	8b, 8c, 9a1, 9a2, 9b
C13	1	3b
C12	2	day7, 7a
C11	1	3c
C10	1	7b
C9	1	2d
C8	1	5b
C7	1	day6
C6	1	4a
C5	1	day3
C4	2	1b, 2c
C3	1	3a
C2	1	1d
C1	2	0b, 1a

Detailed version of cranial OSA network (Figure 3a) labeled with semaphoronts and events. Observed semaphoronts are represented by open ellipses, with width proportional to frequency support, with unobserved semaphoronts reconstructed by OSA are represented by filled nodes. The modal sequences are shown by bold segments. Ambiguity resulted in semaphoronts with multiple possible maturities represented by double-dashed lines.

Figure S3. Detailed forelimb OSA Network

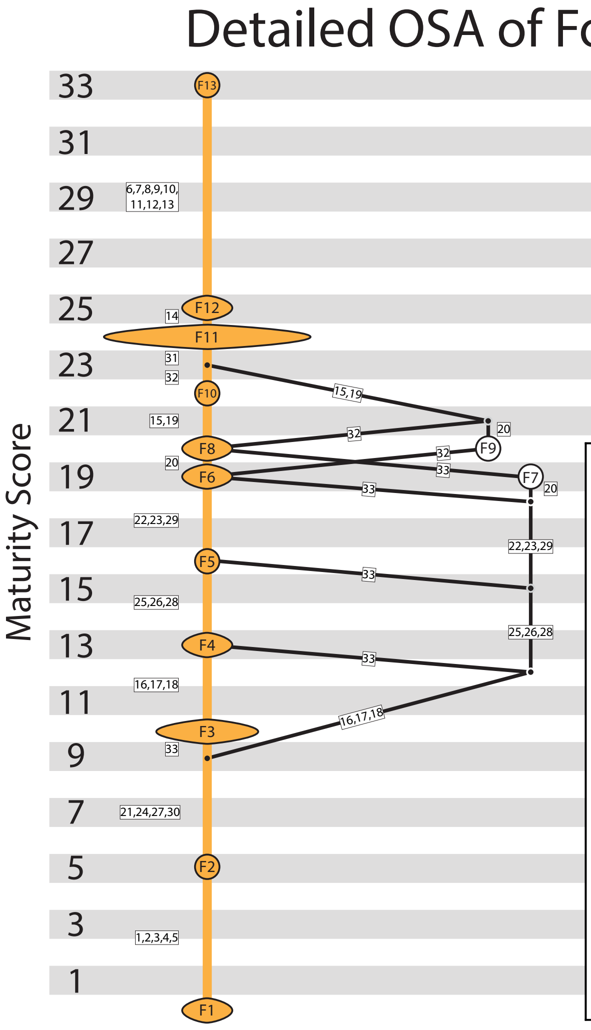

Semaphoront	Frequency Support	Specimen IDs
F13	n/a	hypothetical fully mature specimen
F12	2	20a, 21b
F11	14	9a1, 9b, 10a, 11a, 11b, 13a, 15a, 15b, 15d, 15e, 15f, 15g1, 16a, 17b
F10	1	9a2
F9	1	day8
F8	2	7c, 8b
F7	1	7a
F6	2	7b, 8a
F5	1	5b
F4	2	day7, 8c
F3	9	1b, 1d, 2c, 2d, 3a, 3b, 3c, day3, 4a
F2	1	day6
F1	2	0b, 1a

Detailed version of cranial OSA network (Figure 3b) labeled with semaphoronts and events. Observed semaphoronts are represented by open ellipses, with width proportional to frequency support, with unobserved semaphoronts reconstructed by OSA are represented by filled nodes. The modal sequences are shown by bold segments. Ambiguity resulted in semaphoronts with multiple possible maturities represented by double-dashed lines.

Figure S4. Detailed hindlimb OSA Network

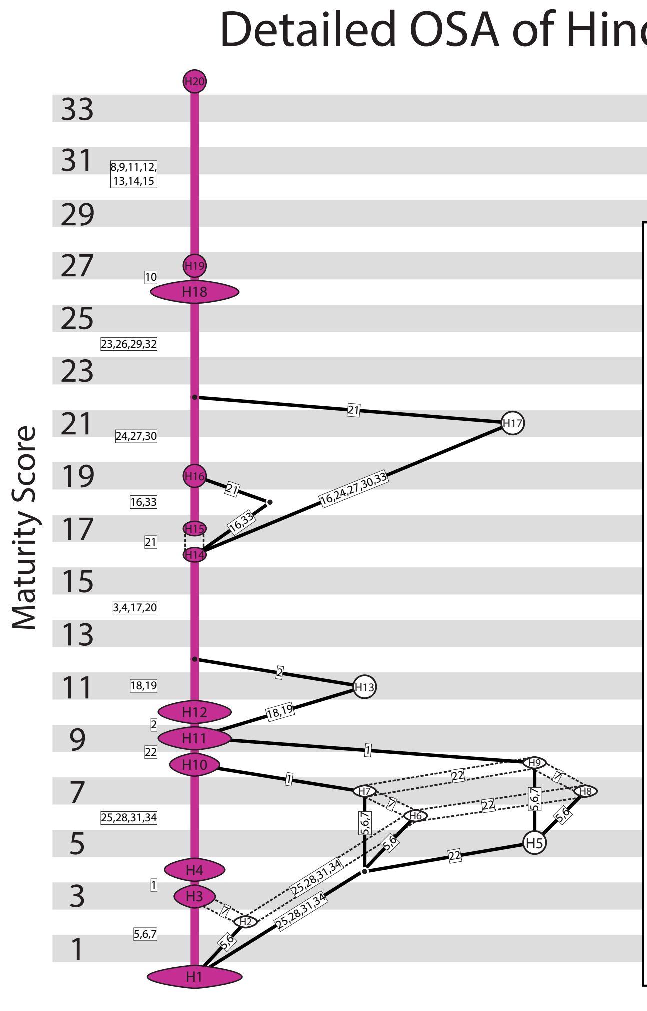

Semaphoront	Frequency Support	Specimen IDs
H20	n/a	hypothetical fully mature specimen
H19	1	21b
H18	7	15a, 15b, 15d, 15g1, 16a, 17b, 20a
H17	1	15f
H16	1	13a
H15	0.5	15e
H14	0.5	15e
H13	1	8c
H12	5	9a1, 9b, 10a, 11a, 11b
H11	5	3c, 7c, 8a, day8, 9a2
H10	2	8b, 7b
H9	0.1667	day6
H8	0.1667	day6
H7	0.1667	day6
H6	0.1667	day6
H5	1	day3
H4	3.3333	3b, 5b, 7a, day7
H3	1.5	3b, 4a, day6
H2	0.1667	day6
H1	7.3333	0b, 1a, 1b, 1d, 2c, 2d, 3a, 3b

Detailed version of cranial OSA network (Figure 3c) labeled with semaphoronts and events. Observed semaphoronts are represented by open ellipses, with width proportional to frequency support, with unobserved semaphoronts reconstructed by OSA are represented by filled nodes. The modal sequences are shown by bold segments. Ambiguity resulted in semaphoronts with multiple possible maturities represented by double-dashed lines.
